# Lymphatic muscle cells are the innate pacemaker cells regulating mouse lymphatic collecting vessel contractions

**DOI:** 10.1101/2023.08.24.554619

**Published:** 2023-10-25

**Authors:** S.D. Zawieja, G.A. Pea, S.E. Broyhill, K.H. Bromert, C. E. Norton, H. J. Kim, M. Li, J.A. Castorena-Gonzalez, B.T. Drumm, M.J. Davis

**Affiliations:** 1 Dept. of Medical Pharmacology & Physiology, University of Missouri, Columbia, Missouri; 2 Dept. of Pharmacology, Tulane University, New Orleans, Louisiana; 3 Smooth Muscle Research Centre, Dundalk Institute of Technology, Dundalk, Co. Louth, A91 K584, Ireland.

**Keywords:** Lymphatic collecting vessel, lymphatic muscle cell, pacemaking, interstitial cells of Cajal like cells, mesenchymal stem cells

## Abstract

Collecting lymphatic vessels (cLVs) exhibit spontaneous contractions with a pressure-dependent frequency, but the identity of the lymphatic pacemaker cell is still debated. By analogy to pacemakers in the GI and lower urinary tracts, proposed cLV pacemaker cells include interstitial cells of Cajal like cells (ICLC), pericytes, as well as the lymphatic muscle (LMCs) cells themselves. Here we tested the extent to which these cell types are invested into the mouse cLV wall and if any cell type exhibited morphological and functional processes characteristic of pacemaker cells: a contiguous network; spontaneous Ca^2+^ transients; and depolarization-induced propagated contractions. We employed inducible Cre (iCre) mouse models routinely used to target these specific cell populations including: c-kitCreER^*T2*^ to target ICLC; *PdgfrβCreER*^*T2*^ to target pericytes; *PdgfrαCreER*^*™*^ to target CD34^+^ adventitial fibroblast-like cells or ICLC; and *Myh11CreER*^*T2*^ to target LMCs. These specific inducible Cre lines were crossed to the fluorescent reporter ROSA26mT/mG, the genetically encoded Ca^2+^ sensor GCaMP6f, and the light-activated cation channel rhodopsin2 (ChR2). c-KitCreER^*T2*^ labeled both a sparse population of LECs and round adventitial cells that responded to the mast cell activator compound 48–80. *PdgfrβCreER*^*T2*^ drove recombination in both adventitial cells and LMCs, limiting its power to discriminate a pericyte specific population. *PdgfrαCreER*^*™*^ labeled a large population of interconnected, oak leaf-shaped cells primarily along the adventitial surface of the vessel. Titrated induction of the smooth muscle-specific *Myh11CreER*^*T2*^ revealed a LMC population with heterogeneous morphology. Only LMCs consistently, but heterogeneously, displayed spontaneous Ca^2+^ events during the diastolic period of the contraction cycle, and whose frequency was modulated in a pressure-dependent manner. Optogenetic depolarization through the expression of ChR2 by *Myh11CreER*^*T2*^, but not *PdgfrαCreER*^*™*^ or c-KitCreER^*T2*^, resulted in a propagated contraction. These findings support the conclusion that LMCs, or a subset of LMCs, are responsible for mouse cLV pacemaking.

## Introduction

The spontaneous contractions of collecting lymphatic vessels (cLV) are an integral component to fluid and macromolecule homeostasis as they provide the force to transport fluid from the interstitium back to the blood circulation (Scallan et al., 2016). In humans, spontaneous contractile activity is estimated to account for 2/3 of lymph transport (Engeset et al., 1977) and this function is significantly compromised in patients suffering from lymphedema, whose cLVs typically display weak and irregular or entirely absent contractile activity (Olszewski, 2002). *Ex vivo* studies, in which the intraluminal pressure can be precisely controlled, have refined our understanding of the pressure-dependent regulation of contraction frequency (Benoit et al., 1989; Gashev et al., 2004), with some mouse cLVs displaying a 10-fold increase in contraction frequency over a 10 cmH_2_O pressure gradient (Scallan and Davis, 2013; Zawieja et al., 2018). The observation that cLVs, often cannulated at various lengths for *ex vivo* preparations, retain a consistently tunable contraction frequency points to the presence of a pacemaker cell(s) innate to the structure of the cLV wall and with a seemingly ubiquitous presence along the length of the vessel (Zawieja et al., 1993; Castorena-Gonzalez et al., 2018b).

Investigations into the cLV pacemaker identity have focused largely on cells termed interstitial cells of Cajal like cells (ICLC; or telocytes) (McCloskey et al., 2002; Briggs Boedtkjer et al., 2013), as they display some morphological and cell marker expression profiles similar to the interstitial cells of Cajal (ICC), which are bona fide pacemakers in the gastrointestinal (GI) tract. ICC are classically identified by either methylene blue staining and expression of cKit, and coordinate GI smooth muscle contraction (Maeda et al., 1992; Ward et al., 1994; Ordog et al., 1999). ICC also express the canonical Ca^2+^ activated chloride channel Anoctamin 1 (Ano1) (Gomez-Pinilla et al., 2009), which is required for the electrical slow wave initiation and formation that subsequently activates an action potential in GI smooth muscle (Hwang et al., 2009; Zhu et al., 2009; Singh et al., 2014). Previous reports in sheep mesenteric lymphatic vessels identified a population of cKit^+^, vimentin^+^, ICLC in the vessel wall between the endothelial and LMC layer and running along the axis of the vessel (McCloskey et al., 2002). Investigations in the human thoracic duct also identified a significant population of ICLC in close proximity to the lymphatic muscle cells (LMCs) evident by methylene blue staining, immunostaining for CD34, vimentin, and cKit, as well as the gold standard of electron microscopy (Briggs Boedtkjer et al., 2013). However, neither study could determine if these cells had functional electrical communication with the LMCs or demonstrate either a membrane electrical clock or internal Ca^2+^ clock to drive the rhythmic lymphatic vessel contractions observed *ex vivo*. LMCs share a functional similarity to ICC in that they also display a Ca^2+^ activated chloride current (Van Helden, 1993; Toland et al., 2000; Mohanakumar et al., 2018), mediated by Ano1 (Zawieja et al., 2019), that regulates pacemaking by modulating the slope of diastolic depolarization. Spontaneous transient depolarizations, presumably Ano1 dependent, were recorded in mesenteric cLVs from guinea pigs (Van Helden, 1993; von der Weid et al., 2008) providing a mechanism for membrane potential instability. Furthermore, computational models have proposed LMC sarcoplasmic reticulum (SR) Ca^2+^ release as the oscillator mechanism driving pacemaking (Imtiaz et al., 2007). SR Ca^2+^ release has also been implicated in pericyte regulation of arterioles (Hashitani et al., 2015; van Helden and Imtiaz, 2019), in microvascular vasomotion (Boedtkjer et al., 2008; Aalkjaer et al., 2011; van Helden and Imtiaz, 2019), and in the contraction waves of atypical muscle cells of the lower urinary tract (Grainger et al., 2022).

Presently, no investigations have clearly identified the cellular identity of possible pacemaker cells within the cLVs of the mouse. Murine cLVs exhibit contractile parameters and conduction speed similar to those of human vessels (Castorena-Gonzalez et al., 2018b) and their simplified architecture, compared to larger mammals, in combination with the genetic tools developed for the mouse model, allowed us to test for a fundamental pacemaker cell in the cLV. In this study we utilized multiple genetic mouse models, immunofluorescence imaging, Ca^2+^ imaging, and optogenetic light-activated depolarization to both visualize and test the functional aspects of putative pacemaker cells. We did not observe a significant or contiguous cKit^+^ cell population along the vessels. *PdgfrαCreER*^*™*^ was able to consistently delineate a significant population of interconnected adventitial cells that were also CD34^+^. However, an absence of Ca^2+^ events in phase with the contractile activity and an inability to consistently elicit a contraction upon photo-stimulated depolarization provides functional evidence against a role for either PDGFRα^+^ or cKit^+^ adventitial cells as the pacemaker cell. In contrast, MYH11^+^ LMCs exhibited diastolic Ca^2+^ events that, while asynchronous, were dynamically modulated by pressure and could elicit a propagated contraction after optogenetic stimulation of LMCs expressing ChR2.

## Results

### Methylene Blue Staining Reveals adventitial Cells in Murine cLVs

**Figure 1:**
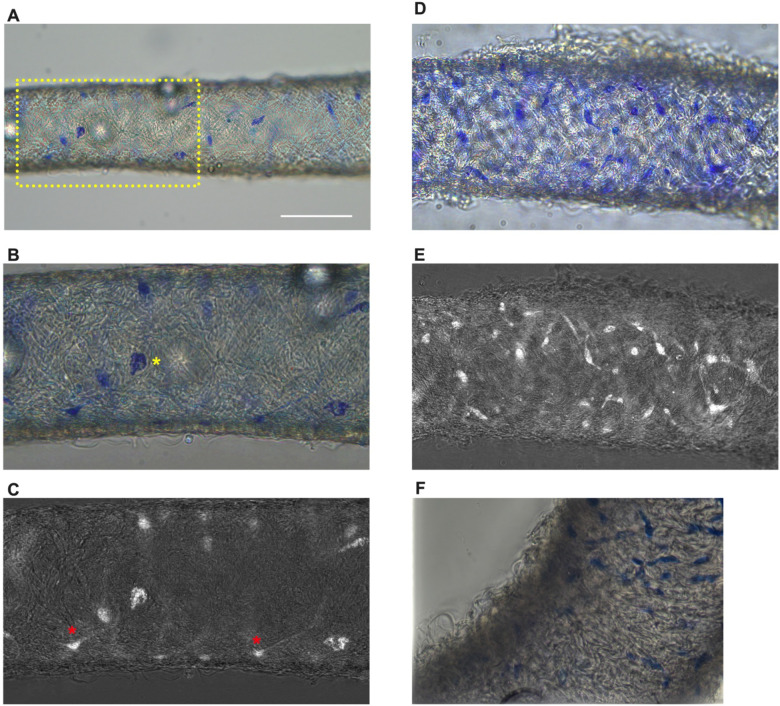
Methylene blue staining of isolated mouse IALVs (A) Representative image of an isolated and cleaned IALV after methylene blue staining which revealed cells of various morphology. (B) is the zoomed in image of the yellow dotted box in A which contained large ovoid cells with granular staining (B, yellow asterisks). Fine cellular extensions (red asterisks) stained by methylene blue in some cells were visualized with color channel separation and division (C). (D, E) Similar as B and C, but in a separate vessel which stained with a higher density of methylene blue stained cells some of which had limited cellular processes. F) Focal reconstruction from imaging a methylene blue stained vessel using an upright microscope and immersion objective.

Methylene blue staining was used to identify an ICLC population in the human lymphatic thoracic duct (Briggs Boedtkjer et al., 2013). In our isolated and cleaned IALVs ,methylene blue stained a significant number of cells along the length of the vessel. The methylene blue^+^ cells had variable density along the length of the vessel and stained cells exhibited different patterns of morphology and location (Figure 1A–C). A significant portion of the stained cells resembled lymphatic vessel-associated macrophages with an elongated spindle shape, while other cells were smaller and circular (Figure 1D–F). Methylene blue also appeared to stain mast cells as there were large ovoid cells on the adventitia of the vessel with intracellular granules. In addition, methylene blue stained a minor population of cells that exhibited long and thin axon-like extensions which appeared to have a slight helical orientation, with a small central body and nucleus (Figure 1C). None of these cell populations were aligned with the longitudinal axis of the vessel that would permit efficient coupling or regulation across the circumferential LMCs required for coordinated propagation along the length of the vessel.

### Immunofluorescence Imaging of IALVs stained for ICLC, LEC, and LMC Markers

**Figure 2:**
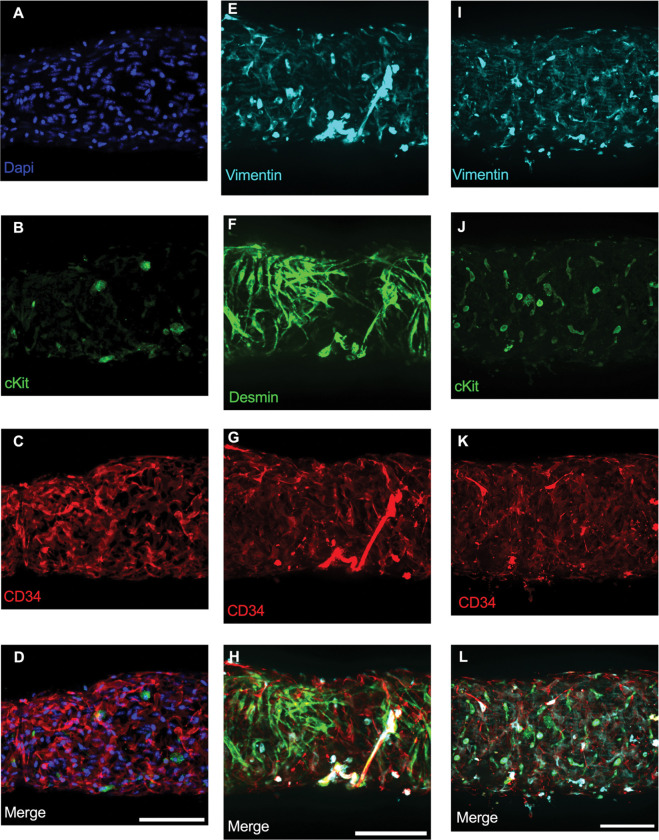
Staining Murine IALVs for ICLC Markers Representative immunofluorescent max projections of half vessel confocal image stacks imaged from mouse IALVs stained for ICLC markers. DAPI (A), cKit (B), and CD34 (C) and their merged image (D). Representative max projections of the intermediate filament vimentin (E), the intermediate filament desmin (F), CD34 (G) and their merged image (H). Representative max projection of vimentin (I), cKit (J), CD34 (K) and their merged image (L). Scale bar = 100 μm for all images.

We first stained IALVs for the putative telocyte/ICLC markers cKit, CD34, and the intermediate filament vimentin, which have been previously described in human and sheep lymphatic tissues (McCloskey et al., 2002; Briggs Boedtkjer et al., 2013). Lymphatic muscle cells also express the intermediate filament desmin (McCloskey et al., 2002). IALVs stained for cKit (Figure 2B) had robust signal in large ovoid cells characteristic of mast cells, confirmed to contain a nucleus via DAPI staining (Figure 2A), that were located in the outer part of the adventitia. Similarly, cKit stained populations of elongated cells as well as circular cells with variable density throughout the IALV wall, similar to methylene blue^+^ cell populations (Figure 2B, J). Staining for CD34 revealed a striking population of cells that were seemingly contiguous along the length of the vessel. These CD34^+^ cells generally had multiple lobular processes and a “oak leaf” like appearance, characteristic of fibroblasts, while some contained short, thin dendrite-like extensions (Figure 2C, G, K). CD34^+^ cells were negative for desmin (Figure 2H), which stained the circumferential LMCs (Figure 2F; note that the largely non-circumferential cell organization in this region is typical for a lymphatic endothelial valve site). Furthermore, the CD34^+^ cells and cKit^+^ stained separate populations (Figure 2D, L). Vimentin stained lymphatic endothelial cells (LECs) which exhibited a horizontal cobblestone morphology in parallel with the vessel axis (Figure 2E, I), while also co-labeling the majority of the CD34^+^ cells (Figure 2H) and cKit^+^ cells (Figure 2L). Videos of the half vessel z-stacks are provided (Supplemental Movies 1–3 for Figure 2D, H, and L respectively).

**Figure 3:**
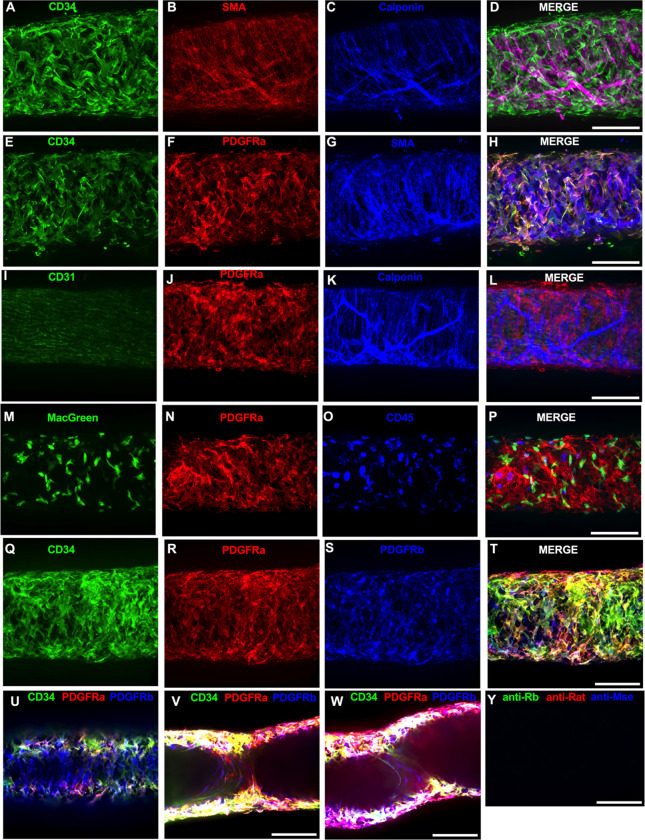
Immunofluorescent Labeling of Murine IALVs With Markers for ICLC, LMC, LEC, and Immune Cell Populations We stained isolated mouse IALVs with cellular markers used to differentiate various cell types observed in cLVs. Half vessel image stacks were taken with confocal microscopy and the resulting representative max projections are shown. (A) CD34 stained cells and LMC staining with SMA (B) and calponin (C) and the corresponding merged (D) image. Significant overlap in (E) CD34 staining along with the fibroblast marker PDGFRα compared to LMC staining with SMA (G) and the merged (H) image. The endothelial marker CD31 (I) to delineate LECs with PDGFRα staining (J), and the LMC marker calponin (K) with the merged image (L). Mononuclear phagocyte cells were identified by anti-GFP (M) in IALVs from MacGreen mice and counter stained with PDGFRα (N) and the hematopoietic marker CD45 (O) with (P) the merged image. PDGFRβ (S) stained many cells that were CD34 (Q) and PDGFRα (R) positive in addition to signal detected in the LMC layer (U). Max projections of only the luminal frames of a z-stack at lymphatic valve locations revealed PDGFRβ, CD34, and PDGFRα labeling in bipolar shaped cells with long extensions that traveled throughout the valve leaflets (V, W). Secondary only stained control IALV (Y). Scale bar = 100 μm for all images.

Of the cells stained in Figure 2, the CD34^+^ population was intriguing due to its high density and seemingly consistent presence through the length of the IALV, which potentially would be conducive to effective regulation of LMC excitability. In addition to CD34 and vimentin, PDGFRα expression is also commonly ascribed to both telocytes (Vannucchi et al., 2013; Xiao et al., 2013; Zhou et al., 2015) as well as fibroblasts (Kimura et al., 2021; Clayton et al., 2022). We performed immunofluorescence imaging for PGDFRα counterstained with CD34 and markers for LMCs, LECs, or myeloid-derived cells. As noted with desmin staining in Figure 2, CD34^+^ cells (Figure 3A) did not co-label LMCs (Figure 3D) that were smooth muscle actin^+^ (Figure 3B) and calponin^+^ (Figure 3C). However, nearly all CD34^+^ (Figure 3E) cells were also PDGFRα^+^ (Figure 3G, H) but not SMA+ (Figure 3G, H). Staining to outline LECs with CD31 (PECAM, Figure 3I) revealed the expected rectangular cobblestone morphology that was distinct from the PDGFRα labeled cells (Figure 3J, L). Calponin specifically stained LMCs (Figure 3K) but not PDGFRα+ cells (Figure 3L). To determine if the CD34^+^PDGFRα^+^ cells had hematopoietic or mononuclear phagocytic origin, we stained IALVs from Macgreen mice (Csfr1-EGFP) with CD45 and PDGFRα. Anti-GFP (Figure 3M) labeled the cLV myeloid cells (Macgreen), which consists of predominantly macrophages and dendritic cells, that were co-stained by CD45^+^ (Figure 3O, P). CD45^+^ but GFP- negative circular cells were also observed and are likely T-Cells, B-cells, or mast cells. As expected, the PDGFRα^+^ cell population (Figure 3N) was not CD45^+^ (Figure 3P). Lastly, we stained for PDGFRα, CD34, and PDGFRβ, a commonly used pericyte marker (Figure 3 Q–T). As above, CD34 and PDGFRα were highly colocalized (Figure 3Q, R, T), and many of the CD34^+^ and PDGFRα^+^ cells were also PDGFRβ^+^ (Figure 3T). PDGFRβ also stained some circumferential LMCs, albeit with a weaker signal than the adventitial cells (Figure 3U). During the imaging of mouse IALVs for these markers, we also observed that the lymphatic secondary endothelial valves were populated by elongated cells that stretched the length of the valve leaflet and were positive for CD34, PDGFRα, and PDGFRβ, with varying intensities, and found within both leaflets of the valve (Figure 3V,W) as determined from max projections of a z-stack encompassing only the “z slices” within the lumen of the IALV. These cells had long, thin extensions that were branched along with apparent dendrite extensions with a morphology that closely resembled those described of pericytes or telocytes (Popescu and Faussone-Pellegrini, 2010) (Figure 3V,W). PDGFRα^+^ or CD34^+^ cells with this morphology were only observed in the valve leaflets and thus seemed insufficient to regulate pacemaking as normal contractions are observed in cLVs without secondary valves (not shown). The z-stacks demonstrating these valve-located “telocyte” shaped cells (Figure 3V,W) are provided as Supplemental Movies 4 and 5.

**Figure 4:**
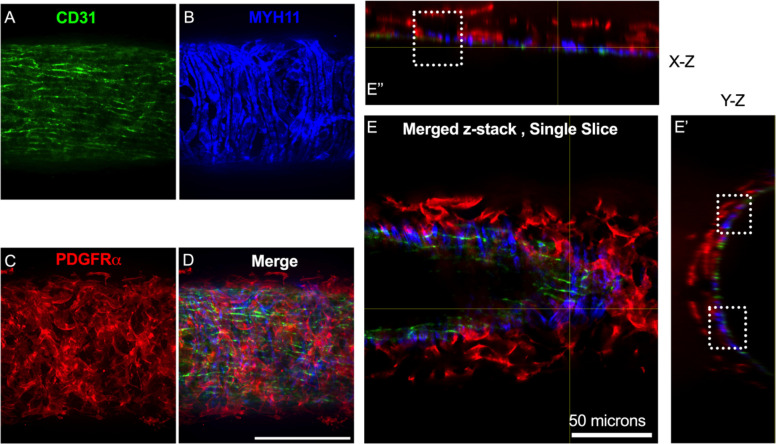
PDGFRα^+^ Cells Reside Primarily in the Murine Lymphatic Collecting Vessel Adventitia and Some in the Subendothelial Space Max projection of confocal imaging of an IALV stained for LECs with CD31 (A), LMCs with MYH11(B), and for PDGFRα (C) with the corresponding merge file (D). Orthogonal views of the z-stack with (E) showing a single slice in the z stack and E’ and E” the orthogonal views. White dotted boxes outline locations where PDGFRα signal is observed between LMC and LEC layers. Scale bar is 100 μm in (D) and 50 μm in (E).

The vast majority of the PDGFRα^+^ cells were located in the adventitial layer Figure 4A–D, which varied between 2–3 PDGFRα^+^ cells thick (Figure 4E) for this particular mouse lymphatic collecting vessel. Under this layer, we observed only a single layer of largely circumferential LMCs stained by MYH11 (Figure 4B) sitting atop a single layer of CD31^+^ LECs (Figure 4A). We also observed occasional PDGFRα^+^ cells or their extensions located in the sub-endothelial space (Figure 4 E’, E”) positioned between the LECs and the LMCs.

**Figure 5:**
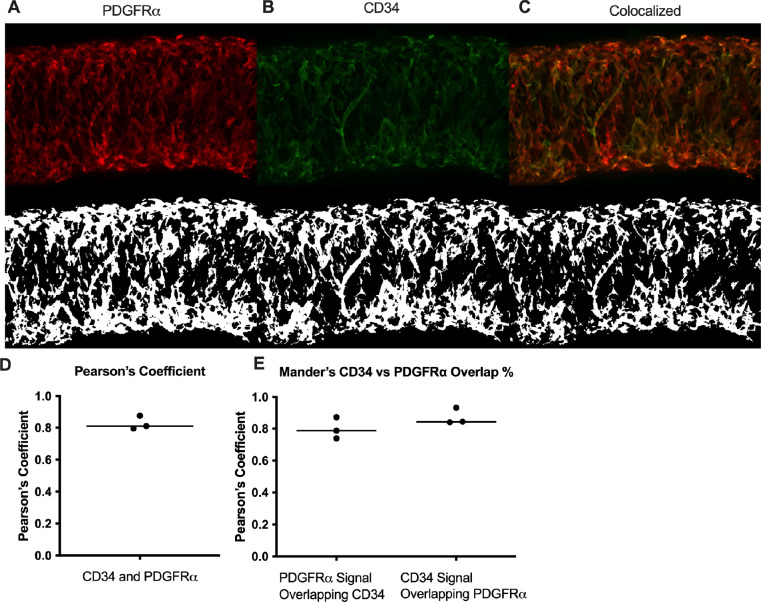
Colocalization of CD34 and PDGFRα Representative max projections and their corresponding threshold adjusted images for colocalization analysis for PDGFRα (A), CD34 (B), and their colocalized signal (C). Pearson’s coefficient and Mander’s coefficients were calculated from 3 separate stained IALVS, each from a separate mouse.

We next determined the degree of colocalization between the CD34 and PDGFRα signal given the significant overlap. Colocalization analysis of PDGFRα (Figure 5A) and CD34 (Figure 5B) and their colocalization (Figure 5C) was determined with the FIJI BIOP-JACoP tool. The Pearson’s coefficient was 0.83 (Figure 5 D) and Mander’s coefficient of overlap 0.80 was for the PDGFRα^+^ signal and 0.87 for the CD34 signal (Figure 5E). This high degree of colocalization CD34 and PDGFRα signal informed our use of the commercially available *PdgfrαCreER*^*™*^ mouse model to target these cells.

### Use of iCre-Mediated Recombination of Rosa26mT/mG to delineate and characterize specific IALV cell types

**Figure 6:**
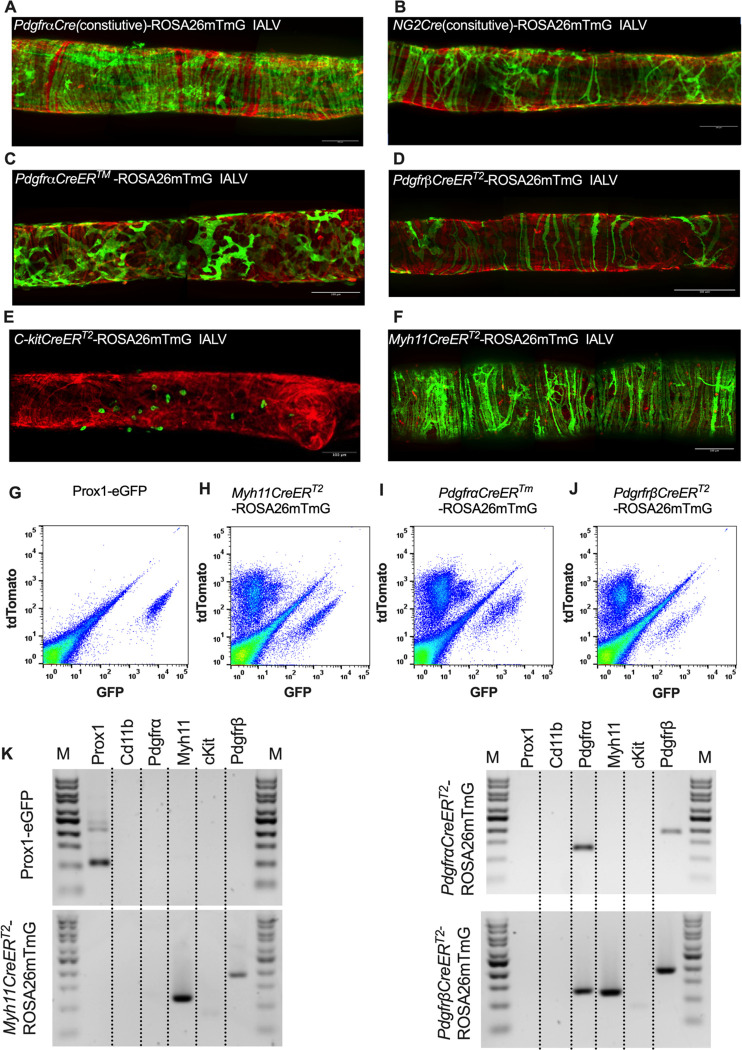
iCre-ROSA26mTmG Labelling and Fidelity to Target Putative Pacemaker Cell Populations Stitched montages of serial max projections of GFP and tdTomato signal from live IALVs isolated from *PdgfrαCre*-ROSA26mTmG (A), *Ng2Cre*-ROSA26mTmG (B), *PdgfrαCreER*^*™*^-ROSA26mTmG (C), *PdgfrβCreER*^*T2*^-ROSA26mTmG (D), *cKitCreER*^*T2*^-ROSA26mTmG (E), and *Myh11CreER*^*T2*^-ROSA26mTmG (F). IALVs were digested into single cells and GFP^+^ cells were purified via FACS from *Prox1-eGFP* (G), *Myh11CreER*^*T2*^-ROSA26mTmG (H), *PdgfrαCreER*^*™*^-ROSA26mTmG (I), and *PdgfrβCreER*^*T2*^-ROSA26mTmG (J) mice. Representative gels demonstrating RT-PCR products corresponding to the respective gene used in the promoter of the transgenes to drive GFP signal or Cre mediated recombination of ROSA26mTmG from each GFP^+^ sorted population (K) to assess fidelity. Images are representative of IALVs from at least 3 separate mice. FACs and RT-PCR was repeated at least 3 times for each mouse.

After confirming the presence of Vimentin^+^, cKit^+^, and CD34^+^ PDGFRα^+^ positive cells within the mouse IALV, we sought to further investigate these cell populations by using constitutive and inducible Cre recombinase expressing mouse lines. IALVs from the constitutively active *PdgfrαCre*-ROSA26mTmG and *Ng2Cre*-ROSA26mTmG mice had GFP fluorescence in the majority of LMCs as well as in the fibroblast-shaped cells found within the IALV wall (Figure 6 A,B). While informative of expression of the LMC progenitor cells, neither constitutive Cre would be useful in delineating cell types. In contrast to the constitutively active *PdgfrαCre*, the tamoxifen inducible *PdgfrαCre*^*™*^ line drove significant recombination in only the fibroblast-shaped cells previously stained with CD34 and PDGFRα but not LMCs or LECs (Figure 6C). *PdgfrβCreER*^*T2*^, commonly used to label pericytes, drove recombination in both a minor population of the LMCs and the fibroblast-shaped cells. *cKitCreER*^*T2*^, which capably drives recombination in the ICC of the GI (Baker et al., 2016), drove recombination only in a small population of irregularly-spaced large ovoid cells on the surface of the IALV (Figure 6E), largely corresponding with mast cell labeling, although recombination in 1 or 2 LECs could occasionally be detected (not shown). Finally, *Myh11CreER*^*T2*^ drove recombination in nearly all LMCs which were largely circumferentially oriented with dendrite-like, cell-cell contacts visible between them and without significant GFP fluorescence in either LECs or the fibroblast-shaped CD34^+^ PDGFRα^+^ cell population (Figure 6F). Additionally, some LMCs maintained the bipolar shape but had multiple extensions forming a “Y” shape in which an adjacent LMC typically filled the inner void. A very minor population of recombined cells in the *Myh11CreER*^*T2*^-ROSA26mTmG IALVs were smaller and irregularly patterned with multiple fine axon-like projections or highly-dendritic ruffled edges (Figure 6F).

To complement the morphological and cell density findings obtained with confocal microscopy, we digested IALVs from the iCre-ROSA26mTmG lines, and the *Prox1-eGFP* line as a control, into single cell suspensions and sorted the respective GFP^+^ populations (Figure 6G–J) for RT-PCR profiling (Figure 6K). We first focused on determining the molecular fidelity of the sorted cells based on the gene promoters used to drive each “iCre” model to discern cellular overlap. In agreement with the confocal images, sorted GFP^+^ cells from *PdgfrβCreER*^*T2*^-ROSA26mT/mG IALVs expressed PDGFRβ but also MYH11 and PDGFRα. In contrast, GFP-sorted cells from *PdgfrαCreER*^*™*^ IALVs expressed PDGFRα and PDGFRβ, but with no detectable expression of MYH11. Conversely, GFP^+^ cells from sorted *Myh11CreER*^*T2*^-ROSA26mTmG IALVs had high expression for MYH11, as well as PDGFRβ, but did not express PDGFRα. IALVs from *cKitCreER*^*T2*^-ROSA26mTmG mice were not pursued for FACS due to the exceptionally sparse recombination along the IALV.

**Figure 7:**
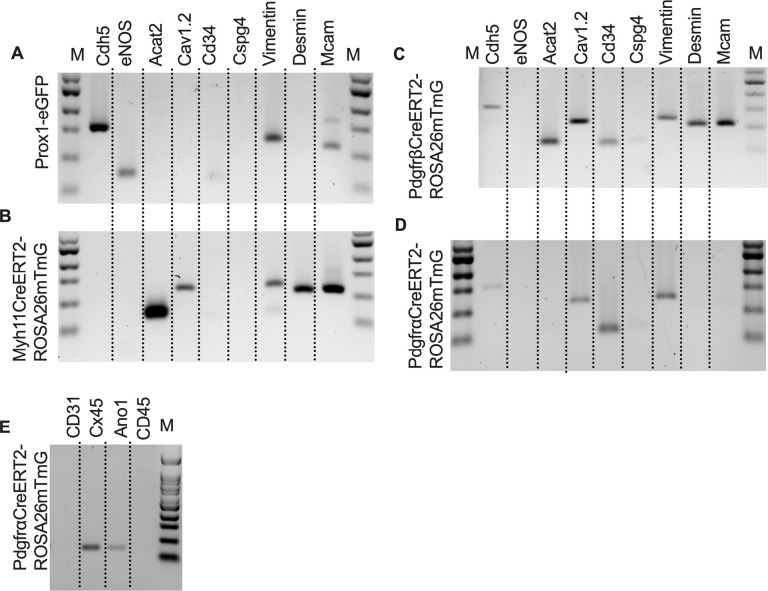
RT-PCR Profiling of FACs Sorted Cells from iCre-ROSA26mTmG Expanded RT-PCR profiling of genes to discriminate LECs, LMCs, and other cell types in our GFP^+^ sorted cells from *Prox1-eGFP* (A), *Myh11CreER*^*T2*^-ROSA26mTmG (B), *PdgfrβCreER*^*T2*^-ROSA26mTmG (C), and *PdgfrαCreER*^*™*^-ROSA26mTmG (D). RT-PCR results for CD31, Cx45, Ano1, and CD45 in GFP^+^ cells sorted from *PdgfrαCreER*^*™*^-ROSA26mTmG IALVs. RT-PCR was repeated at 2–4 times for each gene over each sorted cell population collected from different mice.

We further profiled each population of sorted cells with RT-PCR for other common markers for endothelial cells, muscle cells, and pericytes. Similar to the Prox1 results (Figure 6K), endothelial nitric oxide synthase (eNOS) expression was observed only in the *Prox1-eGFP* sorted cells, which also expressed vimentin and MCAM and had weak but detectable signal for CD34 (Figure 7A). *Myh11CreER*^*T2*^ sorted cells showed expression of smooth muscle actin (Acta2), the alpha subunit of the L-type voltage gated Ca^2+^ channel Cav1.2, desmin, MCAM, and vimentin (Figure 7B). In addition to the genes expressed under *Myh11CreER*^*T2*^ recombination, Cdh5, CD34, and Cspg4 (Ng2) were detected in cells sorted in *PdgfrβCreER*^*T2*^ IALVs (Figure 7C). As expected, the GFP^+^ cells sorted from *PdgfrαCreER*^*™*^ IALVs expressed mRNA for CD34, Cspg4, and vimentin, but not desmin, smooth muscle actin, nor the pericyte marker MCAM (Figure 7D). The alpha subunit of the voltage gated Ca^2+^ channel was positive in cells sorted from both PDGFRα, PDGFRβ,and *Myh11CreER*^*T2*^ IALVs, but was also observed in the *PdgfrαCreER*^*™*^ IALVs without any evidence that MYH11 expressing muscle cells contaminated the latter. These findings confirmed the separate cell populations achieved with *PdgfrαCre*^*™*^ and *Myh11CreER*^*T2*^ mediated recombination, at least as it pertains to ROSA26mTmG. We followed up the identification of Cav1.2 expression in the *PdgfrαCreER*^*™*^ sorted cell population by assessing the expression of other genes involved in either pacemaking (Ano1) or electrical conduction (Cx45) of IALVs (Figure 7E). Intriguingly, expression of both Ano1 and Cx45 was observed in the *PdgfrαCreER*^*™*^ sorted cells, which were further confirmed to lack either endothelial or hematopoietic contamination as we did not detect expression of the endothelial marker CD31 or the hematopoietic marker CD45.

### Inducible Deletion of Either Cav1.2, Ano1, and Cx45 with *PdgfrαCreER*^*™*^ Did Not Affect cLV Pacemaking

**Figure 8:**
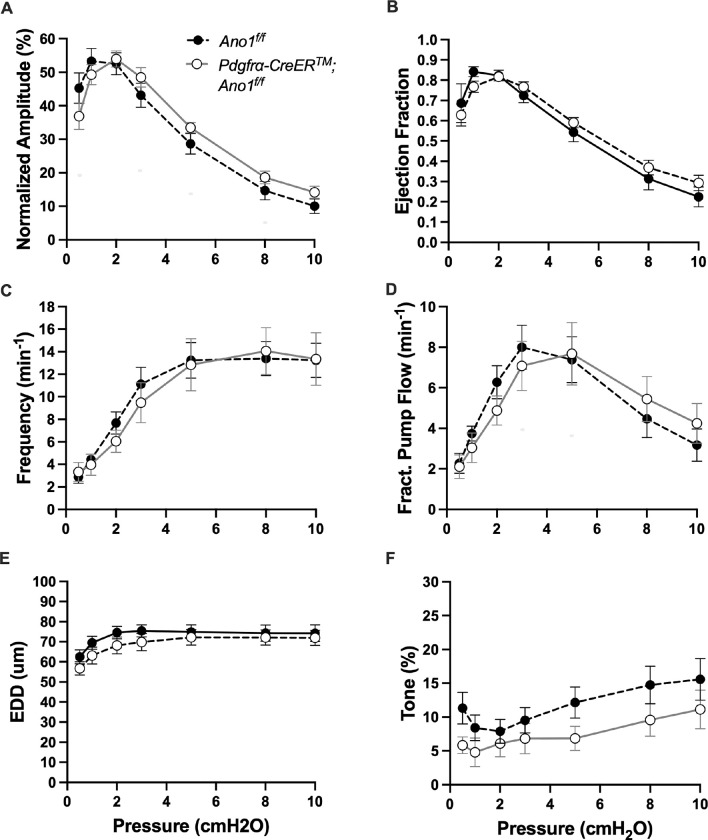
Isobaric contractile Assessment of popliteal cLV from *PdgfrαCre*TM-Ano1 fl/fl mice Summary of the contractile parameters recorded from popliteal cLVs in *PdgfrαCreER*^*™*^-Ano1fl/fl mice. Normalized contraction amplitude (A), ejection fraction (B), contraction frequency (C), fractional pump flow (D), end diastolic diameter (E), and vessel tone (F) were assessed. No statically significant differences observed. Mean and SEM shown, n=6 popliteal vessels from 3 mice *PdgfrαCreER*^*™*^-Ano1fl/fl mice and n=10 popliteal vessels from 6 mice Ano1fl/fl mice.

**Figure 9:**
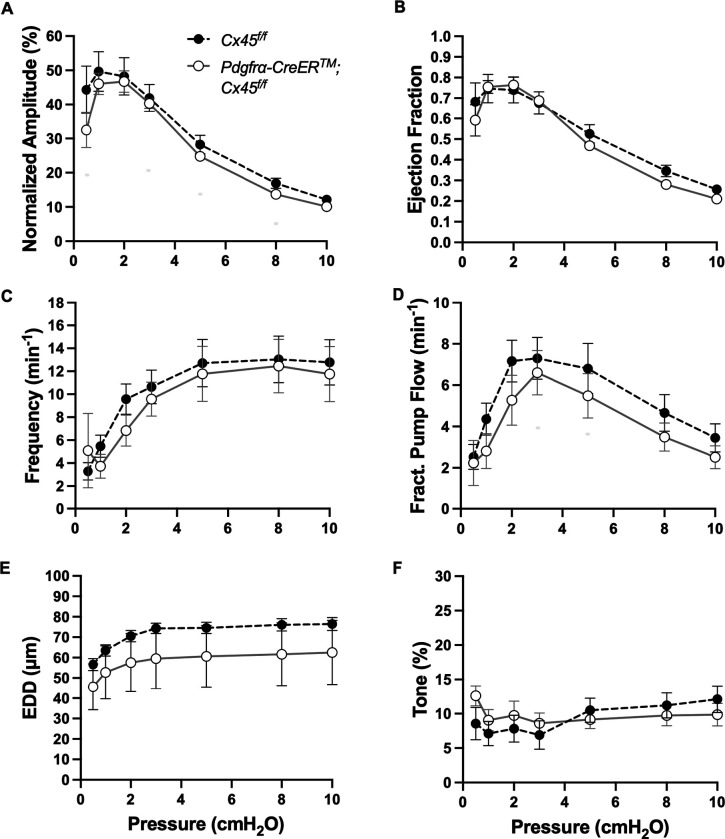
Isobaric contractile Assessment of popliteal cLV from *PdgfrαCre*TM-Cx45 fl/fl mice Summary of the contractile parameters recorded from popliteal cLVs in *PdgfrαCreER*^*™*^-CX45fl/fl mice. Normalized contraction amplitude (A), ejection fraction (B), contraction frequency (C), fractional pump flow (D), end diastolic diameter (E), and vessel tone (F) were assessed. No statically significant differences observed. Mean and SEM shown, n=5 popliteal vessels from 3 mice *PdgfrαCreER*^*™*^-CX45fl/fl mice and n=8 popliteal vessels from 11 mice CX45fl/fl mice.

**Figure 10:**
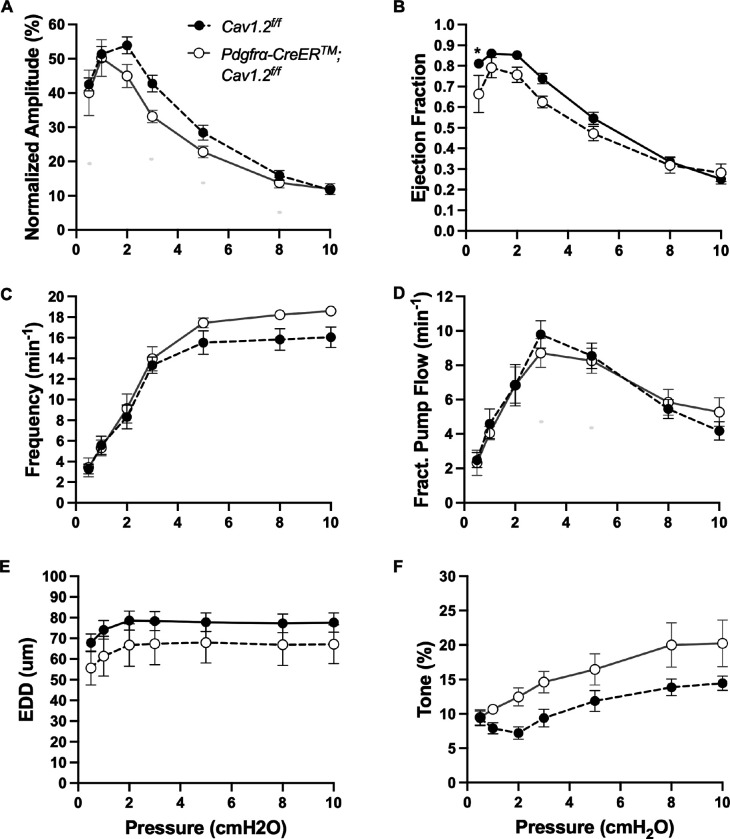
Isobaric contractile Assessment of popliteal cLV from *PdgfrαCre*TM-Cav1.2 fl/fl mice Summary of the contractile parameters recorded from popliteal cLVs in *PdgfrαCreER*^*™*^-Cav1.2fl/fl mice. Normalized contraction amplitude (A), ejection fraction (B), contraction frequency (C), fractional pump flow (D), end diastolic diameter (E), and vessel tone (F) were assessed. Mean and SEM shown, n=6 popliteal vessels from 3 mice *PdgfrαCreER*^*™*^-Cav1.2fl/fl mice and n=9 popliteal vessels from 20 mice Cav1.2fl/fl mice. The contractile data from control Cav1.2fl/fl vessels was previously published but was separated by sex (Davis et al., 2022) while they are combined here. * denotes significance at p <0.05.

The expression of the genes critically involved in cLV function—Cav1.2, Ano1, and Cx45—n the *PdgfrαCreER*^*™*^-ROSA26mTmG purified cells prompted us to generate *PdgfrαCreER*^*™*^-Ano1fl/fl, *PdgfrαCreER*^*™*^-Cx45fl/fl, and *PdgfrαCreER*^*™*^-Cav1.2fl/fl mice for functional testing. We isolated popliteal cLVs and tested their pacemaker and contractile activity in response to physiological pressures over the range 0.5–10 cmH_2_O, under normal conditions. However, we did not detect any significant difference in pacemaking nor contractile function of popliteal cLVs studied from *PdgfrαCreER*^*™*^-Ano1fl/fl (Figure 8A–F) or *PdgfrαCreER*^*™*^-Cx45fl/fl (Figure 9A–F), compared to their respective Ano1fl/f and Cx45fl/fl controls. *PdgfrαCreER*^*™*^-Cav1.2fl/fl mice had no statistically significant differences in normalized contraction amplitude (Figure 10A), contraction frequency (Figure 10C), fractional pump flow (Figure 10D), end diastolic diameter (Figure 10E). However, we noted a mild but statistically significant increase in ejection fraction at the lowest pressure, 0.5 cmH_2_O. Vessels isolated from *PdgfrαCreER*^*™*^-Cav1.2fl/fl mice also had a statistically significant increase in vessel tone (Figure 10F) noted at the 2-way ANOVA level although we did not resolve significance at any specific pressure with this sample size. Nonetheless, despite the presence of transcript for these critical genes, *PdgfrαCreER*^*™*^ mediated deletion failed to recapitulate previous reports of contractile defects using the *Myh11CreER*^*T2*^ line (Castorena-Gonzalez et al., 2018b; Zawieja et al., 2019; To et al., 2020; Davis et al., 2022).

### Potential Role for PDGFRα^+^ Adventitial Fibroblasts As Pool of Progenitor Cells

**Figure 11:**
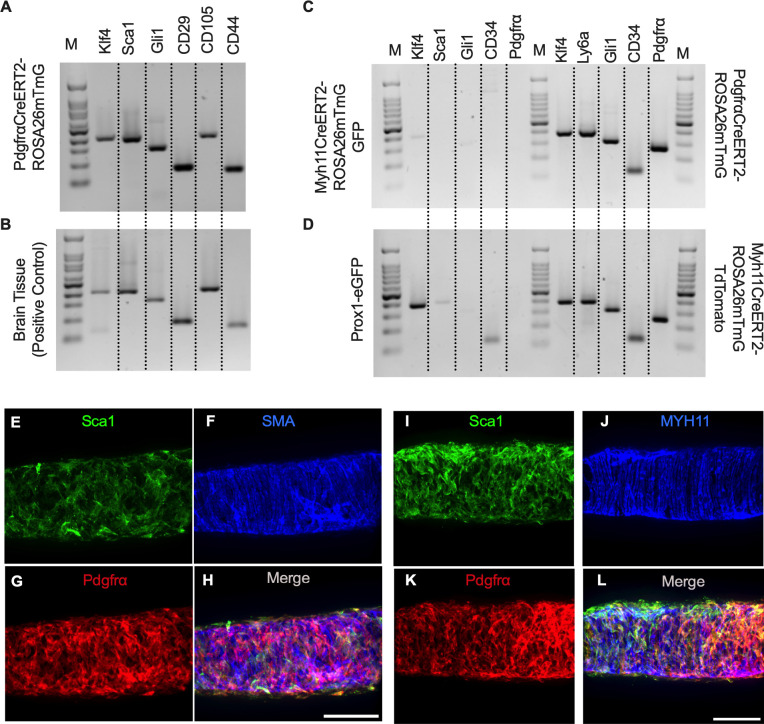
PDGFRα Cells Express Multipotent Cell Makers Representative RT-PCR results profiling purified GFP+ cells purified from IALVs isolated from *PdgfrαCreER*TM-ROSA26mTmG via FACS. PDGFRα cells expressed the multipotent markers Klf4, Sca1, Gli1, CD29, CD105, and CD44 (A) with total brain cDNA serving as a positive control (B). Representative RT-PCR results showing lack of expression of some of these markers in the GFP^+^ cells purified from *Myh11CreER*^*T2*^--ROSA26mTmG (C) or *Prox1-eGFP* mice, in contrast to the RFP^+^ population from *Myh11CreER*^*T2*^--ROSA26mTmG mice (D). RT-PCRs were repeated at least 2 times from separate purified cells populations from different mice. Representative max projections of IALVs stained for Sca1 (E), PDGFRα (E), SMA (F) or Sca1 (I), PDGFRα (K), MYH11 (J), and their corresponding merged file (H, L). Scale bar is 100 μm.

Despite the lack of cLV pacemaking deficits in the *PdgfrαCreER*^*™*^ genetic knockout lines, we were curious to discern further insight into the role or function of the PDGFRα^+^ CD34^+^ cells which comprise a significant portion of the lymphatic cLV wall. The *PdgfrαCreER*^*™*^ recombined cells exhibited expression of Krüppel-like factor 4 (Klf4), stem cell antigen 1 (Sca1, also referred to as Ly6a), Gli1, CD29, CD105, and CD44 (Figure 11A, B). To again validate that this signature was unique to the *PdgfrαCreER*^*™*^ recombined cells, we performed RT-PCR on FACS from Prox1-eGFP, *Myh11CreER*^*T2*^-ROSA26mTmG, and *PdgfrαCreER*^*™*^-ROSA26mTmG IALVs. Recombined (GFP^+^) cells from *Myh11CreER*^*T2*^-ROSA26mTmG had weak expression of Klf4 and Gli1 and were negative for Sca1, CD34, and PDGFRα (Figure 11C). Similarly, LECs sorted from *Prox1-eGFP* IALVs were positive for Klf4, weak for Sca1, and positive for CD34 but negative for GLi1 and PDGFRα Gli1. In contrast, *PdgfrαCreER*^*™*^- ROSA26mTmG recombined cells were positive for all markers as were the unrecombined population (tdTomato^+^) cells in the *Myh11CreER*^*T2*^- ROSA26mTmG IALVs (Figure 11D). We performed immunofluorescence staining for one of these multipotent markers, Sca1 (Figure 11E, I) while counter staining for LMCs, with SMA or MYH11 (Figure 11F, J), and the adventitial cells with PDGFRα (Figure 11G, K). The morphology and staining pattern of Sca1 overlapped significantly with PDGFRα staining and not the LMC staining (Figure 11H, L, Supplemental Movie 6).

### Optogenetic Stimulation of iCre-driven Channel Rhodopsin 2 to Induce Test Light-Stimulated Depolarization Induced Lymphatic Contraction

**Figure 12:**
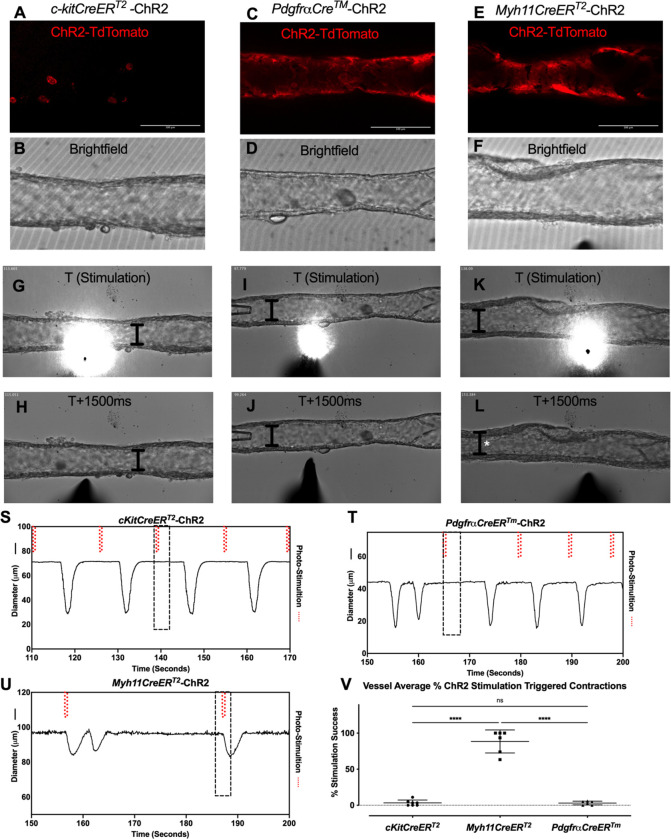
ChR2-Mediated Depolarization in LMCs Only Triggers Contraction Representative max projections of tdTomato-ChR2 signal in popliteal cLVs isolated from *cKitCreER*^*T2*^-ChR2-tdTomato (A), *PdgfrαCreER*^*™*^-ChR2-tdTomato (C), and *Myh11CreER*^*T2*^- ChR2-tdTomato (E) with their corresponding brightfield image (B, D, F) respectively. Time-lapse brightfield images every 0.5 s starting at stimulation t=0 for *cKitCreER*^*T2*^-ChR2-tdTomato (G-J), *PdgfrαCreER*^*™*^-ChR2-tdTomato (K-N), and *Myh11CreER*^*T2*^- ChR2-tdTomato (O-R). The I bar denotes the inner diameter at t=0 over time and white asterisks denote the contraction. Representative diameter trace for the popliteal cLV demonstrate spontaneous contractions with the dotted boxes indicating the optical stimulation event in the respective brightfield images of the time lapse images. Isolated cLVs from *cKitCreER*^*T2*^-ChR2-tdTomato (S), *PdgfrαCreER*^*™*^-ChR2-tdTomato (T), and *Myh11CreER*^*T2*^- ChR2-tdTomato (U) were stimulated with light pulses (red dashed lines) and the summation of contraction triggering for each genotype (V). Mean and SEM are shown, **** denotes p<0.0001. Contraction recorded from at least 6 popliteal cLVs from 3 mice per genotype.

We next used optogenetic methods to test whether the cell populations recombined by either *cKitCreER*^*T2*^, *PdgfrαCreER*^*™*^, or *Myh11CreER*^*T2*^ could elicit a coordinated contraction. The ChR2-tdTomato construct appeared more sensitive to recombination than ROSA26mTmG, in some cases resulting in LMC expression of ChR2-tdTomato in *PdgfrαCreER*^*™*^ and *CKitCreER*^*T2*^ vessels. Care was taken to image the vessel for tdTomato (Figure 12A,C,E) prior to stimulation at their respective sites under brightfield conditions for diameter tracking (Figure 12B,D,F) to ensure fidelity of the cell types and morphologies observed in Figure 3. As with ROSA26mTmG, *CKitCreER*^*T2*^ drove the ChR2-tdTomato expression primarily in large ovoid cells found on the adventitia of the vessel. Photo-stimulation of these cells did not initiate coordinated contractions (Figure 12G–J,S). Similarly, photo-stimulation of ChR2-tdTomato expressing cells driven by *PdgfrαCreER*^*™*^ failed to initiate a coordinated contraction (Figure 12K–N, T). In contrast, photo-stimulation of LMCs, using *Myh11CreER*T2 to express Chr2-tdTomato, resulted in a propagated contraction in the popliteal vessel (Figure 12O–R, U). In total, only 3.25% of photo-stimulations of *cKitCreER*^*T2*^-ChR2-TdTomato and 3.03% of photo-stimulations of *PdgfrαCreER*^*™*^-ChR2-tdTomato were associated with a contraction, while 88.5% of photo-stimulations of *Myh11CreER*^*T2*^-ChR2-tdTomato photo-stimulations induced contractions (Figure 12V). The low percentages of optogenetic firing of contractions observed in *PdgfrαCreER*^*™*^-ChR2-tdTomato and *cKitCreER*^*T2*^-ChR2-TdTomato vessels are likely due to the happenstance of spontaneous contractions occurring during the time and proximity of optogenetic stimulation. As mast cells are not ascribed any tissue specific pacemaking behavior, the similar percentages observed between these two groups is suggestive of coincidence. Brightfield videos of the photo-stimulation and representative traces for *cKitCreER*^*T2*^-ChR2-TdTomato, *PdgfrαCreER*^*™*^-ChR2-tdTomato, *Myh11CreER*^*T2*^-ChR2-tdTomato are provided in Supplemental Movies 7–9.

### Confocal Ca^2+^ Imaging of GCaMP6f Expression Driven by *cKitCreER*^*T2*^, *PdgfrαCreER*^*™*^, and *Myh11CreER*^*T2*^ Over the Lymphatic Contraction Cycle

**Figure 13:**
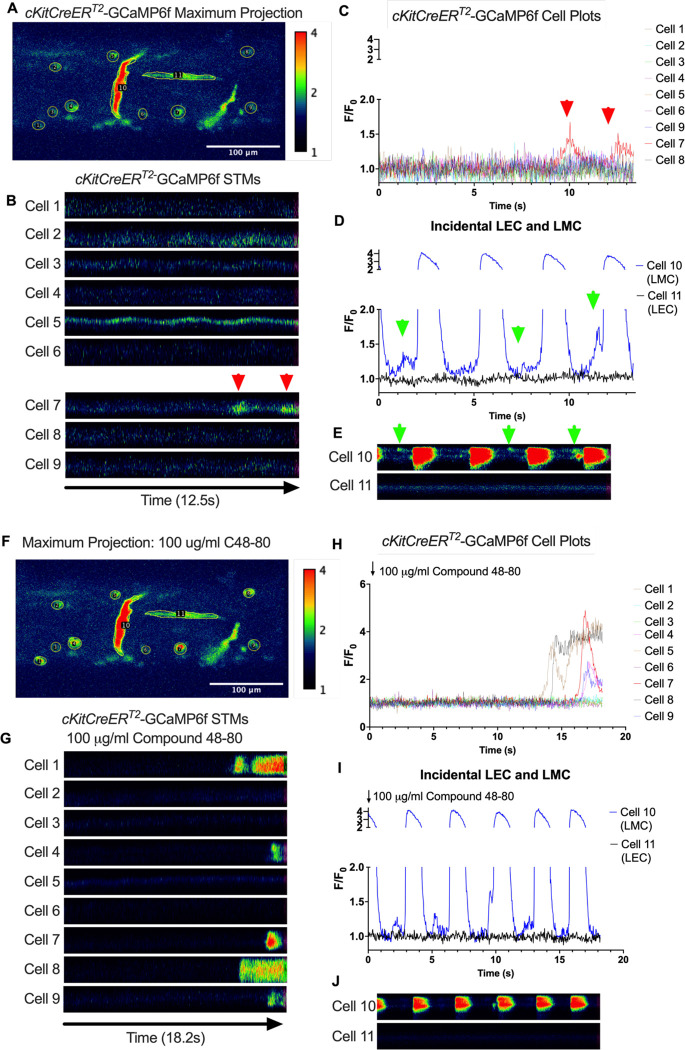
*cKitCreER*T2 Drives GCaMP6f Expression in Primarily Mast Cells in Murine IALVs Representative max projection of GCaMP6f signal over time in an IALV isolated from a *cKitCreER*^*T2*^-GCaMP6f mouse with ROI indicated around individual cells, primarily large ovoid cells, but also including a circumferential LMC (Cell10) and a horizontal LEC (Cell 11). Of cells 1–9, only cell 7 had any Ca^2+^ activity (red arrows) during the recording time as indicated by the STMs from each ROI (B) and their normalized F/F_0_ plots in (C). In contrast, the LMC in ROI 10 had both rhythmic global Ca^2+^ events (D) that spanned the cell axis (vertical axis) in the STM (E) in addition to localized Ca^2+^ events intervening the time between global events (green arrows). Representative max projection of GCaMP6f signal over time after stimulation with C48–80 (F) with many large ovoid cells displaying long lasting global Ca^2+^ events (G,H) while not immediately affecting the LMC Ca^2+^ dynamics (I,J).

**Figure 14:**
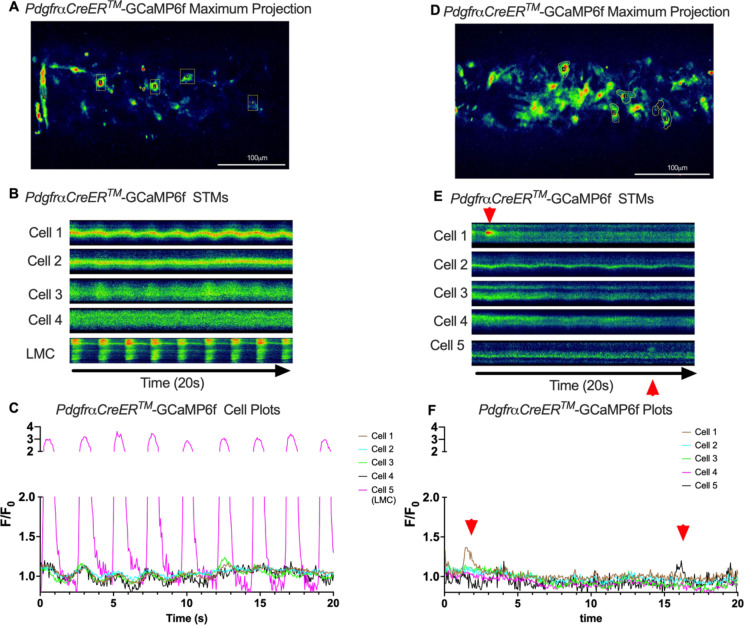
Lack of coordinated Ca^2+^ Activity Across Contraction Cycle in PDGFRα Cells Representative max projections of GCaMP6f signal over time in an IALVs isolated from *PdgfrαCreER*^*™*^-GCaMP6f mice (A, D). ROIs were made around cells and GCaMP6f recorded over time to generate the corresponding STMs (B, E) for each cell and plots (C, F) respectively. Once again, incidental recombination occurred in a LMC which displayed rhythmic Ca^2+^ flashes (C) while the slight undulation in the other cells is due to movement artifact (B). Red arrows indicate the limited local Ca^2+^ activity observed in two cells from a *PdgfrαCreER*^*™*^-GCaMP6f IALV.

**Figure 15:**
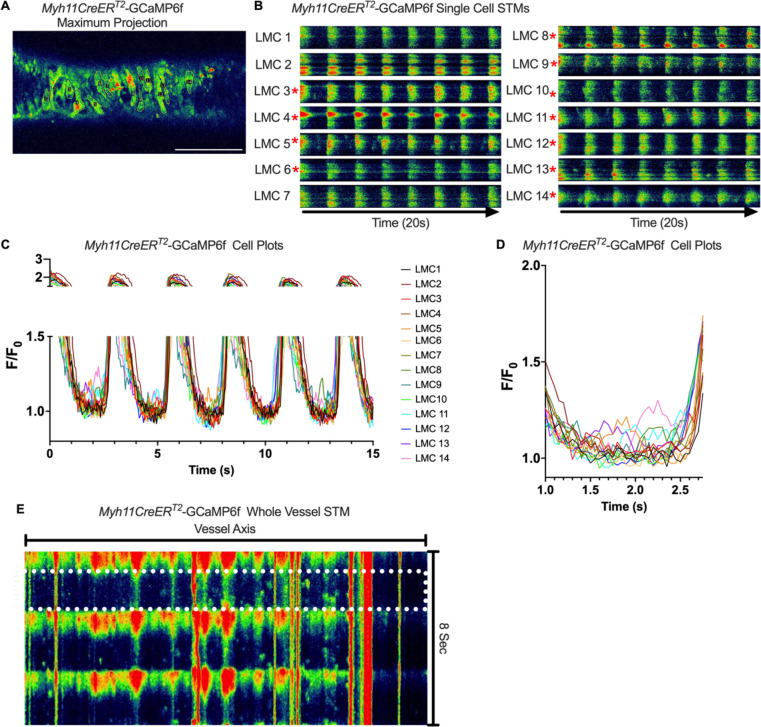
Heterogeneous Diastolic Ca^2+^ Transient Activity in LMCs Representative max projections of GCaMP6f signal over time in an IALVs isolated from *Myh11CreER*^*T2*^-GCaMP6f mice (A). LMCs were outlined with ROIs to assess GCaMp6F signal over time. Rhythmic global flashes (B) were entrained across all the LMCs in the FOV (C) with many cells exhibiting diastolic Ca^2+^ release events. Cells exhibiting at least one diastolic Ca^2+^ event, within the context of our focal plane constraints, over the recorded time were denoted by the red asterisks. The plot in (D) magnifies the first diastolic period, seconds 1–3, of C to assist in visualizing the lack of coordination of the diastolic events. (E) Max projection of the pseudo-linescan analysis across the axis of the vessel to highlight diastolic Ca^2+^ transients in all cells in the field of view and their lack of coordination across the cells (x-axis). The white dotted box shows the first diastolic period plotted in (D).

We imaged IALVs from *cKitCreER*^*T2*^-GCaMp6f mice, which primarily resulted in expression of GCaMp6f in the large ovoid cells in the adventitia (Figure 13A), although we occasionally observed GCaMP6f expression in both LEC and LMCs (Figure 13A) as depicted in the maximum projection of the acquisition period (Supplemental Movie 10) and the spatio-temporal maps (STMS). The aberrant expressions of GCaMP6f in cells that demonstrated the typical cobblestone morphology of LECs or the circumferential LMCs that exhibited Ca^2+^ flashes and diastolic Ca^2+^ transients (Figure 13D,E green arrows) prior to contraction (Figure 13D,E) were not included in the *cKitCreER*^*T2*^-GCaMp6f analysis. Of the 39 *cKitCreER*^*T2*^-GCaMp6f cells analyzed, only 1 *cKitCreER*^*T2*^-GCaMP6f cell exhibited a spontaneous Ca^2+^ transient during the recording period (Figure 13B,C Cell 7). However, the Ca^2+^ transient in that cell did not align temporally with the “Ca^2+^ flash” of the LMC with incidental GCaMp6f expression (Figure 13C,D). Despite the lack of Ca^2+^ transients under the baseline conditions throughout the IALV contraction cycle, many *cKitCreER*^*T2*^-GCaMP6f cells exhibited a robust and prolonged Ca^2+^ event in response to stimulation with the mast cell activator compound 48–80 (Figure 13F, G, H). Notably, the Ca^2+^ events in the ovoid cells elicited by administration of compound 48–80 did not acutely alter the LMC Ca^2+^ activity (Figure 13I,J). Similar to *cKitCreER*^*T2*^-GCaMp6f, the majority of PDFRaCreER^*™*^-GCaMP6f expressing cells also largely lacked Ca^2+^ transients and also resulted in incidental LMC GCaMP6f expression (Figure 14B, Supplemental Movie 11). Some cells exhibited high basal Ca^2+^ (Figure 14A,D) sustained throughout the recording, but oscillations were not observed (Figure 14B,C). In contrast, spurious GCaMP6f expression in a circumferentially oriented LMC displayed Ca^2+^ flashes associated with contraction (Figure 14B,C). Of the 21 PDGFRα-GCaMP6f cells assessed, only 3 exhibited Ca^2+^ transients which were singular events with limited spatial spread within the 20 sec imaging period (Figure 14E,F). The lack of either global or consistent Ca^2+^ transients within either *cKitCreER*^*T2*^-GCaMP6f or *PdgfrαCreER*^*™*^-GCaMP6f IALVs was in stark contrast to Ca^2+^ imaging of *Myh11CreER*^*T2*^-GCaMP6f IALVs. *Myh11CreER*^*T2*^ drove GCaMp6f expression in the circumferential LMCs (Figure 15A), which had global and nearly synchronous Ca^2+^ flashes in 100% of the analyzed cells (Figure 15B, C). Additionally, non-synchronous stochastic and localized Ca^2+^ transients during diastole were commonly observed in the LMCs (Figure 15D, E, Supplemental Movie 12). Many LMCs exhibited Ca^2+^ transients during each diastolic period while other LMCs displayed few Ca^2+^ transients or lacked diastolic Ca^2+^ transients during the recording periods (Figure 15B). In aggregate, of the 39 *cKitCreER*^*T2*^-GCAMP6f cells only 1 displayed a Ca^2+^ transient during recording, 3 of the 21 *PdgfrαCreER*^*™*^-GCaMP6f cells, while 20 of 43 LMCs displayed at least one diastolic transient apart from 43 of 43 LMCs with global flashes.

### Pressure Dependency of Subcellular Ca^2+^ Transients in LMCs

**Figure 16:**
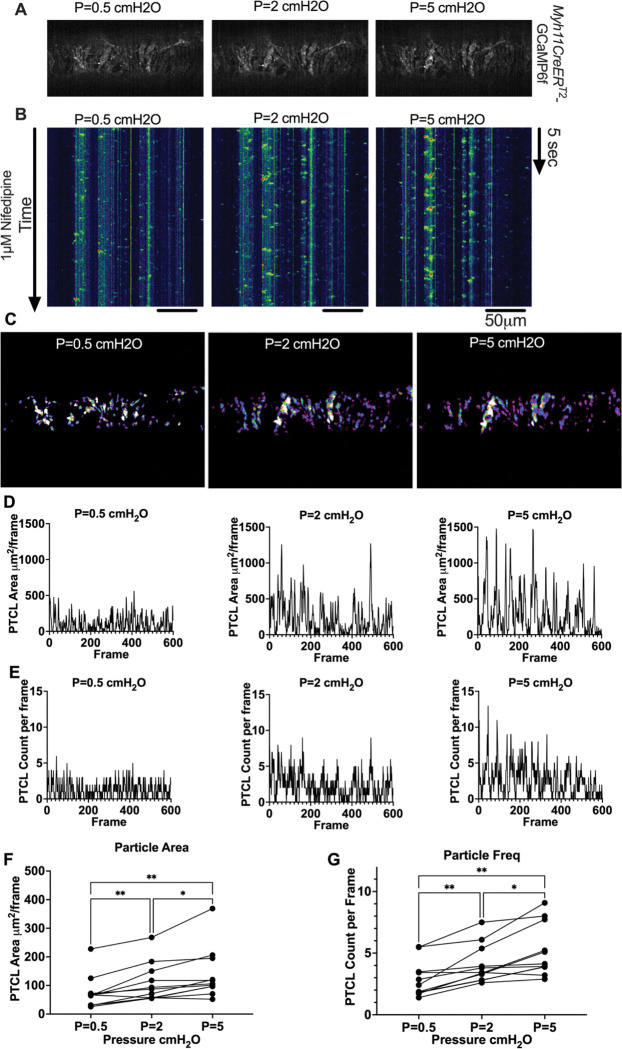
Pressure Dependency of Murine LMC Diastolic Ca^2+^ Transients Representative max projection of GCaMP6f signal over 20 s in an IALVs isolated from *Myh11CreER*^*T2*^-GCaMP6f mice in the presence of the L-type blocker nifedipine (1μM) (A) pressurized to 0.5 cmH_2_O, 2 cmH_2_O, 5 cmH_2_O. The local diastolic Ca^2+^ transients persist in the presence of nifedipine and increase with increasing pressure as demonstrated in the whole vessel STMs (B). Particle occurrence maps highlight the Ca^2+^ activity in each LMC as pressure is raised (C). Representative particle analysis plots for particle area (D) and particle counts/frame at each pressure (E). Summary files for particle area (F) and count /frame (G0. * denotes p<0.05, Mean and SEM shown with n=12 separate IALVs from 8 MYH11-CreER^*T2*^-GCaMP6f mice.

We next sought to test whether diastolic Ca^2+^ transients were pressure-dependent, given that cLVs exhibit pressure dependent chronotropy (Zawieja et al., 2019). GCaMP6f expressing LMCs were studied in the presence of nifedipine, which blocks the calcium flashes but not local calcium transients at intraluminal pressures of 0.5 −5 cmH_2_O (Figure 16A). As intra-luminal pressure was increased, there was a marked increase in the occurrence of Ca^2+^ transients (Figure 16B, Supplemental Movies 13–15). By converting raw Ca^2+^ transients for particle analysis (PTCLs), we generated activity maps of Ca^2+^ PTCL activity (Figure 16C) and determined PTCL area (Figure 16D) and frequency at each pressure (Figure 16E). The maps show that as pressure increased, the activity of PTCLs across the vessel also increased (as evident by the increase in PTCL area activation). Across 11 experiments, the area of the field of view activated by PTCLs/frame increased from 73.2 ± 17.7 mm^2^/frame at 0.5 cmH_2_0 to 108.6 ± 20.5 mm^2^/frame at 2 cm H_2_0 and further enhanced to 139.2 ± 26.9 mm^2^/frame at 5 cm H_2_O (Figure 16F). The number of PTCLs per frame also increased with pressure, from 2.9 ± 0.4 at 0.5 cmH_2_0 to 4.1 ± 0.5 and 5.2 ± 0.6 PTCL/frame at 2 and 5 cmH_2_0 respectively (Figure 16E).

## Discussion

The identification of the cellular origin and signaling mechanisms underlying cLV pacemaking will open up novel targets for pharmacological intervention in treating lymphedema and the associated lymphatic contractile dysfunction. In this study we tested proposed pacemaker cell types based on 3 parameters: 1) that the pacemaker cells are located along the entire length of the cLV, to accommodate spontaneous contractions and coordinated electrical conduction despite progressive shortening of cLVs; 2) that depolarization of the pacemaker cell can drive a coordinated and propagated contraction of the vessel; and 3) that the presence of Ca^2+^ transients precedes contraction, as commonly observed in other pacemaker cell types. We used confocal microscopy and a combination of immunofluorescence and fluorescent reporters under the control of various inducible Cres to identify various non-muscle cells that express the markers CD34 and PDGFRα. From our initial fluorescence imaging studies, a role for intrinsic pacemaking by LMCs (Van Helden, 1993; von der Weid et al., 2008), or by a novel population of lymphatic ICLC (McCloskey et al., 2002; Briggs Boedtkjer et al., 2013), also referred to as telocytes, were further examined. However, the PDGFRα cell population had minimal Ca^2+^ activity and optogenetic depolarization of these cells failed to drive a spontaneous contraction. In contrast, photo-stimulation of LMCs expressing ChR2 elicited robust propagating contractions similar to spontaneous activity. Furthermore, Ca^2+^ imaging in LMCs revealed diastolic Ca^2+^ transients in diastole that increased in frequency and spatial spread as pressure was elevated. Our results, in addition to the recent findings using targeted deletion of Ano1 (Zawieja et al., 2019), Cx45 (Castorena-Gonzalez et al., 2018b), or Cav1.2 (To et al., 2020; Davis et al., 2022) in lymphatic muscle support the model of LMCs as the intrinsic pacemaker as has been previously proposed (Van Helden, 1993; Van Helden et al., 1996; Van Helden and Zhao, 2000).

### Pacemaking in Smooth Muscle

In many smooth muscle organs, regulation of a coordinated contraction is a complex and multicellular phenomenon. Multiple cell types integrate physical and biological information into electrical activity to be transmitted to the force-producing smooth muscle cells, sometimes across great distances relative to cell size, to regulate calcium influx by voltage dependent calcium channels required for contraction. The intestine is one such documented tissue in which cKit^+^ ICCs and interstitial PDGFRα^+^ cells form an electrical syncytium to regulate intestinal motility (Sanders et al., 1999; Sanders et al., 2014). The pacemaking function of intestinal ICCs relies heavily on Ano1, a calcium activated chloride channel, which is required for slow wave activity in the ICCs. Both cKit and Ano1 can be used as a marker for ICCs in the intestine (Hwang et al., 2009; Cobine et al., 2017; Malysz et al., 2017), cKit^+^ and vimentin^+^ ICLCs have been observed in sheep lymphatic vessels (McCloskey et al., 2002), yet these cell populations do not form gap junctions with the smooth muscle to form electrical connections (Briggs Boedtkjer et al., 2013) as occurs in the intestinal ICCs. Our cKit staining of mouse IALVs revealed a sparse population of large ovoid cells previously classified as mast cells (Chatterjee and Gashev, 2012; Zawieja et al., 2019). Their identity as mast cells was further supported by the sustained global Ca^2+^ event after stimulation with the mast cell degranulating agent compound 48–80. However, both vimentin and CD34 showed robust staining throughout the mouse lymphatic vessel wall. Vimentin stained LECs, as well as non-muscle stellate shaped cells, with many co-expressing CD34, and other smaller circular cells some of which were cKit^+^ as well and some whose morphology was similar to that of the macrophage staining profile of the GFP^+^ cells in IALVs from MacGreen mice , as well as previous reports of macrophage staining in cLVs (Bridenbaugh et al., 2013; Chakraborty et al., 2015; Zawieja et al., 2016). While vimentin^+^ cells had a nuclear and perinuclear staining profile, CD34 demarcated the cell membrane and was useful for assessing the morphology of these cells. Of particular interest, the vimentin^+^CD34^+^ cells densely populated the length of the mouse IALV, with a majority displaying a flattened stellate morphology characterized by the classic rounded oak leaf appearance, although some displayed fine dendrite extensions. Contrasting with the previous findings in the human thoracic duct (Briggs Boedtkjer et al., 2013), we did not observe a significant population of CD34^+^ cells with a bipolar morphology oriented axially along the vessel. However, z-stack reconstructions of sections of the mouse IALV that included the secondary valves revealed interstitial CD34^+^PDGFRα^+^ cells that resembled the bipolar cells with multiple axon-like extensions throughout the endothelial leaflets similar to the interstitial cells that were reported in the lymphovenous valves (Geng et al., 2016). While these cells have not been frequently described in the peripheral cLV valves, we observed these cells in each of the valve regions we imaged in addition to labeling them with other Cre drivers, including NG2-Cre;ROSA26mTmG and *PdgfrβCreER*T2 ROSA26mTmG (data not shown). Whether these cells play a role in the extracellular matrix deposition or lymphatic valve integrity is unknown, but their role as a critical pacemaker can be excluded as vessel segments without valves display normal contractile behavior. Instead, the majority of the CD34^+^PDGFRα^+^ cells were found in the adventitia with 2–3 layers overtop the LMCs and were consistently observed in high density along the IALV. Some CD34^+^PDGFRα^+^ cells or their extensions were present between the lymphatic endothelial and muscle layers as had been previously reported with electron microscopy of human lymphatic vessels (Briggs Boedtkjer et al., 2013).

### PDGFRα^+^CD34^+^ Cells are Not Involved in cLV Pacemaking Under Physiological Conditions

CD34 and PDGFRα are described as telocyte markers, although PDGFRα routinely labels fibroblasts and specific interstitial cells in the GI tract involved in purinergic neurotransmission (Kurahashi et al., 2011; Kurahashi et al., 2013; Clayton et al., 2022), and CD34 expression has been observed in multipotent cell populations of various origins (Sidney et al., 2014). Of course, neither telocytes, hematopoietic or mesenchymal stem cells, nor fibroblasts, are monolithic in their expression patterns within and much less across tissues, and single cell RNA sequencing has provided immense detail about the sub clusters and spectrums within which these cells exist as well as their plasticity. Nonetheless, we attempted to gain further insight into the characteristics of CD34^+^PDGFRα^+^ cells. CD34^+^PDGFRα^+^ cells were consistently negative for the majority of smooth muscle markers such as desmin, calponin, smooth muscle actin, smooth muscle myosin heavy chain, and the pericyte marker MCAM. However, PDGFRβ expression was noted in sorted *PdgfrαCreER*^*™*−^ROSA26mTmG cells, in addition to the staining of LMCs, and PDGFRβ protein expression was confirmed with variable immunofluorescence staining amongst the PDGFRα stained cells as well as LMCs. The *PdgfrβCreER*T2-ROSA26mTmG mice had only modest recombination in both the LMC and PDGFRα^+^ cell population, but potentially highlighted a myofibroblast-like cell subpopulation, cells that might lie on the spectrum of differentiation from lymphatic muscle and PDGFRα^+^ cells, or perhaps a cell with pacemaker activity as PDGFRβ is widely used as a pericyte marker and some pericytes display pacemaker activity (Hashitani et al., 2015). Adding to this intrigue, the *PdgfrαCreER*^*™*^ sorted cells expressed transcripts for Cav1.2, the voltage-gated L-type Ca^2+^ channel critical for lymphatic contractions (Zawieja et al., 2018; To et al., 2020); Ano1, the ion channel underlying pressure dependent chronotropy (Mohanakumar et al., 2018; Zawieja et al., 2019); and Cx45, the primary connexin mediating electrical conduction in mouse lymphatic collecting vessels (Castorena-Gonzalez et al., 2018b; Hald et al., 2018). The presence of those gene transcripts does not appear to be due to muscle cell contamination or incidental recombination in LMCs as we did not detect LMC markers in the RT-PCR profiling of the sorted PDGFRα^+^ cells nor were GFP-expressing cells with an LMC morphology observed in imaging of *PdgfrαCreER*^*™*^-ROSA26mTmG vessels. Critically, however, deletion of Cav1.2, Cx45, or Ano1 through *PdgfrαCreER*^*™*^ mediated recombination neither recapitulated the previous phenotypes achieved with *Myh11CreER*^*T2*^ (Castorena-Gonzalez et al., 2018b; Zawieja et al., 2019; To et al., 2020; Davis et al., 2022) nor significantly affected pacemaking in mouse popliteal cLVs. This is in stark contrast to the complete lack of contractions observed in *Myh11CreER*T2-Cav1.2 vessels (To et al., 2020) or the vessels from vascular muscle specific Itga8CreERT2-Cav1.2fl/fl mice (Davis et al., 2022; Warthi et al., 2022),and the significant loss in pressure-induced chronotropic modulation of pacemaker function in IALVs with *Myh11CreER*^*T2*^-mediated deletion of Ano1 that we have previously reported (Zawieja et al., 2019) . While a sub-population of CD34^+^PDGFRα^+^ cells may share expression of critical pacemaker genes identified in the LMCs, they do not appear to be involved in cLV pacemaking or contractile function under physiological states. Instead, CD34^+^PDGFRα^+^ cells co-stained significantly with Sca1^+^, suggesting they may be primed to act as resident multipotent cells (Song et al., 2020; Kimura et al., 2021). To this point, the *PdgfrαCreER*^*™*^ FACS purified cells also expressed markers associated with “stemness” such as CD34, Klf4, Gli1, CD29, CD105, CD44, and vimentin, in addition to Sca1, and it is likely that the *PdgfrαCreER*^*™*^ population includes various distinct subpopulations (Jolly et al., 2022) expressing these markers. These cells may play a role in rebuilding the lymphatic collecting vessel vasculature following lymph node resection and further studies are required to assess their functional multipotency.

### SR Ca^2+^ Cycling in Pacemaking

The use of the mouse model, in addition to the simplicity of the vessel architecture, provided the use of genetic tools that previously had been instrumental in identifying the cKit^+^ ICC as the pacemaker cells of the GI tract (Ward et al., 1994; Huizinga et al., 1995; Torihashi et al., 1995). Through the use of the respective *PdgfrαCreER*^*™*^ and *Myh11CreER*^*T2*^ models, were able to specifically image Ca^2+^ in each cell type in pressurized, contracting vessels. Pacemaking initiating cells have an inherently unstable membrane potential, oftentimes utilizing the oscillatory nature of Ca^2+^ release from the sarcoendoplasmic reticulum coupled to Ca^2+^ sensitive electrogenic exchangers and ion channels to drive depolarization (Van Helden, 1993; Hashitani et al., 2015; Baker et al., 2021b; Sanders et al., 2022). One such example is the pacemaker ICC in the gastric corpus which exhibit abundant Ca^2+^ transients that couple to Ano1-mediated chloride currents in both the intervening period between slow waves as well as the plateau phase of the slow wave (Baker et al., 2021a), although such activity is not characteristic of all pacemaker ICC types. The identification of a Ca^2+^ activated chloride current in LMCs (Van Helden, 1993; Toland et al., 2000) and its correspondence with subcellular Ca^2+^ transients (Van Helden, 1993; Ferrusi et al., 2004; von der Weid et al., 2008) led Van Helden to postulate that LMCs had an intrinsic pacemaking capability (Van Helden, 1993; Van Helden et al., 1996). We have previously reported that mouse LMCs in pressurized vessels routinely display subcellular Ca^2+^ release events that reflect the kinetics and characteristics of Ca^2+^ puffs and waves in addition to the coordinated global Ca^2+^ flash associated with influx during an action potential (Castorena-Gonzalez et al., 2018b; Zawieja et al., 2018; Zawieja et al., 2019). Here we confirmed the consistent presence of subcellular Ca^2+^ transients only in LMCs with GCaMP6f driven by *Myh11CreER*^*T2*^ but not in the cells with GCaMP6f driven by *PdgfrαCreER*^*™*^. Critically, we also demonstrated that the Ca^2+^ transients increased in both frequency and spatial spread as pressure was elevated in the vessel, as would be expected to account for the pressure dependent chronotropy observed in lymphatic collecting vessels. This underscores the recent finding that the genetic deletion of Ano1 in the LMCs dramatically reduced contraction frequency and abolished pressure-dependent chronotropy in those vessels (Zawieja et al., 2019). This phenotype was largely replicated with a similar reduction in frequency and loss of pressure dependent chronotropy in our recent study utilizing *Myh11CreER*^*T2*^ to drive deletion of IP3R1 from LMCs (In revision, JGP 2023) and fits with the central role of IP3R and subcellular Ca^2+^ release as critical components of intrinsic LMC pacemaking (Van Helden et al., 1996; von der Weid et al., 2008). The lack of Ca^2+^ transients in PDGFRα^+^ cells across any stage of the lymphatic contraction cycle diminishes any expected role for this cell type to perform as the pacemaker for the mouse IALV. The contribution of specific LMC SR Ca^2+^ signals to lymphatic pacemaking remains to be fully addressed.

A pacemaker cell would be expected to be electrically coupled to the LMC layer to permit the nearly synchronous conduction velocity of the contraction wave (Zawieja et al., 1993; Castorena-Gonzalez et al., 2018b; Hald et al., 2018) and to transmit their own depolarization into their coupled LMCs to activate the voltage dependent Ca^2+^ channels that are responsible for lymphatic muscle action potentials. Connexins are the molecular constituents of gap junctions and, as stated above, we detected Cx45 expression in the *PdgfrαCreER*^*™*^ sorted cells. However, we did not detect any impairment in pacemaking, nor were contraction conduction speed deficits or multiple pacemakers noted in the *PdgfrαCreER*^*™*^ -Cx45fl/fl popliteal cLVs, in contrast to the development of multiple pacemaker sites and the lack of entrainment that were reported previously in *Myh11CreER*^*T2*^-Cx45fl/fl cLVs (Castorena-Gonzalez et al., 2018b). Admittedly, we did not perform an exhaustive assessment of the connexin expression profile of the CD34^+^PDGFRα^+^ cells, and Cx45 may not be the dominant connexin expressed in the CD34^+^PDGFRα^+^ cells, or heterotypic connexons could exist (Koval et al., 2014). However, electron microscopy studies of the putative ICLC in the human thoracic duct did not detect any gap junctions, although peg-and-socket connections were observed (Briggs Boedtkjer et al., 2013). We utilized optogenetics to enforce channel rhodopsin expression in both the *PdgfrαCreER*^*™*^ and *Myh11CreER*^*T2*^ mouse models to directly depolarize the specific cell populations in an attempt to drive a contraction. Local photo-stimulation of the PDGFRα cells failed to initiate contraction while the stimulation of *Myh11CreER*^*T2*^ recombined cells resulted in contractions that were indistinguishable from the spontaneously occurring contractions. These results give functional credence to the lack of hetero-cellular coupling that was previously reported . Just as critically, they also highlight the regenerative nature of the lymphatic muscle action potential as local depolarization was sufficient to drive a coordinated contraction along the vessel and that a single or few LMCs reaching threshold for AP initiation are sufficient to drive the conducted activity observed at the tissue level.

## Conclusions

Our present findings lend further support to the hypothesis that the LMCs are intrinsic pacemakers (van Helden et al., 2006; Mitsui and Hashitani, 2020) and do not require an ICC-like network to drive propagated contractions. These findings also underscore the significance of lymphatic muscle Ca^2+^ handling as the driver of lymphatic pacemaking, which can be compromised in disease states leading to impaired lymphatic contractile activity (Stolarz et al., 2019; Lee et al., 2020; Van et al., 2021). Further studies delineating the specific SR Ca^2+^ release and influx pathways, and the contributions of Ca^2+^ sensitive ion channels need to be identified to develop sophisticated in silico models and identify potential therapeutic targets to rescue lymphatic pacemaking in lymphedema patients (Olszewski, 2002, 2008).

## Limitations

One assumption underlying our conclusions is that there is a conserved fundamental pacemaking pathway in lymphatic muscle pacemaking across species, specifically pertaining to the capability of lymphatic muscle to maintain pacemaking and coordination despite changes in tissue complexity. Lymphatic collecting vessels in mice have similar pressure-dependent chronotropy and contraction conduction velocity as recorded in human vessels (Castorena-Gonzalez et al., 2018b). These similarities exist despite the fact that mouse lymphatic collecting vessels are encircled by a single layer of lymphatic muscle while larger species have multiple layers of LMCs in the wall. It is possible that vessels with multiple layers of LMCs need a network of ICLC to coordinate their activity. The simplicity in the makeup of the mouse cLV and the use of cell targeting Cre models provide great control over our experimental variables, but other cell types may provide coordination of LMC pacemaking in other species where the lymphatic cLV walls are larger with multiple muscle layers.

Our data demonstrates that limited staining of a few cell markers alone is insufficient to identify discrete cell populations in the murine cLVs. Additionally, mRNA expression does not equal protein translation nor guarantee specific function as we did not detect endothelial CD34 with immunofluorescence despite detecting transcript; additionally, *PdgfrαCreER*^*™*^ mediated deletion of Ano1, Cx45, or Cav1.2 had no effect on cLV pacemaking. Hence, further experimentation is also required to fully characterize expression of multipotent cell markers and function of CD34^+^PDGFRα^+^Sca1^+^ cells invested within the murine cLVs, although this was beyond the scope of this study assessing pacemaker identity. Tangentially, another limitation of our approach pertains to the specificity and recombination efficiency of inducible Cre recombinase models, which can be a notable confounding variable (Chakraborty et al., 2019). We observed that our inducible Cre models led to a degree of nonspecific recombination within the murine cLV, with the GCaMP6f and ChR2 particularly susceptible to recombination compared to the ROSA26mT/mG reporter. Recombination in multiple cell types was expected with the constitutive Cre models we employed (*Ng2Cre* and *PdgfrαCre*), as vascular and lymphatic muscle precursor cells can transiently express nestin, PDGFRα, and NG2 (Hill et al., 2015; Castorena-Gonzalez et al., 2018b; Kenney et al., 2020). We also observed that *PdgfrβCreER*^*T2*^ drove recombination in a sub population of LMCs and PDGFRα^+^ cells. Whether these are two distinct populations that only share expression for PDGFRβ or whether they demonstrate a continuum of precursor LMCs and newly-formed LMCs are both plausible explanations. PDGFB-PDGFRβ signaling is critical for normal mural cell recruitment to both the blood and lymphatic vasculature (Gaengel et al., 2009; Wang et al., 2017) and proliferating vascular smooth muscle cells and pericytes have both been documented to express PDGFRβ (Andrae et al., 2008; Pitulescu and Adams, 2014). We have recently performed single cell RNAseq on isolated IALVs and, while these this dataset is still being analyzed, sub-populations of LMCs, LECs, and CD34^+^ and PDGFRα^+^ were readily identified (data not shown) highlighting the difficulty in ascertaining functionality on the basis of expression of a few cell markers. Ideally, novel Cre or combinatorial Cre models may be developed to further tease out the functional role of these expected sub populations.

## Materials and Methods

### Mice

Wild-type (WT) male mice (25–35 g) on the C57BL/6J background , ROSA26mT/mG reporter (Muzumdar et al., 2007) (Strain#007676), transgenic *PdgfrαCre* (Strain#013148), CSFR1-EGFP (MacGreen) (Sasmono et al., 2003) (Strain#018549), genetically encoded Ca^2+^ sensor GCaMP6f (Chen et al., 2013) (Strain#028865), transgenic *PdgfrαCreER*^*™*^ (Kang et al., 2010) (Strain#018280), NG2-Cre (Strain #:008533) (Zhu et al., 2008), and ChR2 /tdTomato fusion mice (Madisen et al., 2012) (Strain#012567) were purchased from The Jackson Laboratory (Bar Harbor, MA, USA). *PdgfrβCreER*^*T2*^ (Gerl et al., 2015) mice were a gift from Ralf Adams (Mac Planck Institute) and kindly provided by Lorin Olson (Oklahoma Medical Research Foundation), and are currently available at Jax (Strain#029684). The *Myh11CreER*^*T2*^ mice (Wirth et al., 2008) were a gift from Stefan Offermanns, Max-Planck-Intstitut fur Herz-und Lungendforschung, Bad Nauheim, Germany, and are currently available at Jax (Strain #019079, Y-Linked). c-KitCreER^*T2*^ mice (Heger et al., 2014) were a gift from Dieter Saur (Technical University of Munich). Prox1-eGFP mice (Choi et al., 2011) were a gift from Young-Kwon Hong (University of Southern California. For genotyping, we isolated genomic DNA from mouse tail clips using the HotSHOT method (Truett et al., 2000). Specific mouse genotypes were confirmed via PCR using 2x PCR Super Master Polymerase Mix (Catalog # B46019, Bimake, Houston, TX) performed as specified by the provider. Mice were over 3–8 months of age during this study. All animal protocols were approved by the University of Missouri Animal Care and Use Committee and conformed to the US Public Health Service policy for the humane care and use of laboratory animals (PHS Policy, 1996).

### iCre Tamoxifen Induction-

Mice harboring *PdgfrαCreER*^*™*^, *PdgfrβCreER*^*T2*^, *Myh11CreER*^*T2*^, and *cKitCreER*^*T2*^ were crossed with ROSA26mT/mG mice to generate *PdgfrαCreER*^*™*^-ROSA26mT/mG, *PdgfrβCreER*^*T2*^-ROSA26mT/mG, *Myh11CreER*^*T2*^-ROSA26mT/mG, and *cKitCreER*^*T2*^-ROSA26mT/mG mice, respectively. The resulting iCre-ROSA26mT/mG mice were induced with tamoxifen 2–4 weeks after weaning. Tamoxifen induction was performed via consecutive 100 μL i.p. injections of tamoxifen ranging from 1 to 5 days at concentration ranging from 0.2 −10 mg/mL in safflower oil, using a titrated induction protocol to determine the extent of recombination in specific cell populations. We used our maximal induction protocol, 100 μL of tamoxifen at 10 mg/mL over 5 consecutive days, for *cKitCreER*^*T2*^-GCaMP6f, *Myh11CreER*^*T2*^-GCaMP6f, and *PdgfrαCreER*^*™*^ - GCaMP6f mice. Due to the paucity of recombined cells in the *cKitCreER*^*T2*^-ROSA26mT/mG reporter mice, we used our maximal tamoxifen induction protocol for *cKitCreER*^*T2*^-ChR2 mice as this still resulted in the ability to excite single recombined cells. *Myh11CreER*^*T2*^-ChR2/tdTomato mice were induced with one 100 μL i.p. injection of tamoxifen at 0.2 mg/mL while *PdgfrαCreER*^*™*^-ChR2/tdTomato were induced with 1 injection at 0.4 mg/mL tamoxifen to get mosaic induction sufficient for single cell stimulation. All mice, regardless of duration, were given 2 weeks to recover following tamoxifen injection.

### Lymphatic Vessel Isolation-

We utilized both popliteal and inguinal-axillary lymphatic collecting vessels (IALVs) in this study, which were isolated as described previously (Zawieja et al., 2018). In brief, mice were anaesthetized with a cocktail of 100/10 mg/mL) ketamine/xylazine mg/mL and shaved along the flank or the legs for IALVs and popliteal cLVs respectively. The IALV (also referred to as the flank cLV) is located adjacent to the thoracoepigastric vein and connects the inguinal and axillary lymph node. A cut was made along the dorsal midline and the skin retracted and pinned out to reveal the thoracoepigastric vascular bed. The thoracoepigastric vascular bed and connected perivascular adipose containing the IALVs vessel was dissected out and pinned onto a Sylgard coated dish in Krebs buffer. Popliteal lymphatic vessels were exposed through a superficial incision in the leg, removed and transferred to the Krebs-albumin filled dissection chamber. After removal, the vessel was carefully cleaned of adipocytes and excess matrix using fine forceps and scissors through micro-dissection. For immunofluorescence, sections containing 2–3 valves were isolated, while smaller IALV sections consisting of 1–2 valves were isolated for GCaMP6f Ca^2+^ imaging. Similarly, popliteal cLVs were isolated (Castorena-Gonzalez et al., 2018a) following an incision along the skin overlying the saphenous vein for contractile function analysis and for ChR2 optogenetic depolarization experiments.

### Lymphatic Vessel Isobaric Function-

*PdgfrαCreER*^*™*^ mice were crossed with Ano1fl/fl, Cx45fl/fl, and Cav1.2fl/fl and to achieve *PdgfrαCreER*^*™*^-Ano1fl/fl, *PdgfrαCreER*^*™*^-Cx45fl/fl, and *PdgfrαCreER*^*™*^-Cav1.2fl/fl mice. Theses mice and their respective fl/fl controls were injected with tamoxifen as described above for 5 days and given two weeks to recover. The popliteal vessels were isolated, cleaned, and prepared for isobaric contractile tests as previously reported (Davis et al., 2023). Once equilibrated, inner diameter was tracked over a physiological pressure range (stepped from 3 to 2, 1, 0.5, 3, 5, 8, and 10 cmH_2_O) with 2min of recording at each pressure. Following the pressure step protocol the vessels were equilibrated in with Ca^2+^-free Krebs buffer (3mM EGTA) and diameter at each pressure recorded under passive conditions. The contractile parameters end diastolic diameter (EDD), end systolic diameter (ESD), and contraction frequency (FREQ) were recorded with a custom LabVIEW program and the following contractile parameters assessed:

1. Contraction Amplitude (AMP) = EDD−ESD
2. Normalized Contraction Amplitude = ((EDD−ESD)/DMAX) × 100
3. Ejection Fraction (EF) = (EDD^2^−ESD^2^)/EDD^2^
4. Fractional Pump Flow (FPF) = EF × FREQ
5. Tone = ((DMAX−EDD)/DMAX) × 100

### Methylene Blue-

Isolated IALVs sections were transferred into a Krebs-BSA buffer filled 3-mL observation chamber, with a cover slip bottom, and cannulated onto two glass micropipettes (30–80 μm, outer diameter) held in place by pipette holders on a Burg-style V-track mounting system. The pipette holders were attached to a 3-way valve stop cock with polyethylene tubing filled with Krebs-BSA buffer. Vessels were pressurized to approximately 5 cmH_2_O by raising the 3-way valve and the vessels were stretched to remove any slack. For methylene blue staining, IALVs from wild type C57Bl6 mice were stained with 50 μM methylene blue in Krebs-BSA buffer for two hours at room temperature and covered in foil to limit light induced phototoxicity. After the staining period, the vessel chambers were washed three times with Ca^2+^ free PSS to remove methylene blue. Brightfield images and manual Z-stack videos were collected on an inverted Leica DMi1 4X or 20X air objective, or a Leica DMi8 with a 25X water objective or an inverted DMi8 using a Leica Flexacam C1 color camera for image acquisition. Some Methylene blue images were also collected using a color Nikon DS-Fi3 camera. The collected z-stacks were analyzed using Image J and the “Stack Focuser” plugin (https://imagej.nih.gov/ij/plugins/stack-focuser.html). To accentuate the methylene blue stained cells, the color image stack was split into red, green, and blue channel stacks. The blue channel stack was then divided by the green channel stack using the “Image Calculator” function. The resulting 32-bit image was then converted into 16-bit image to permit the use of the Stack Focuser plugin with the ‘n kernel value’ set to 11.

### Fluorescence Confocal Imaging-

IALVs vessels from each respective iCre-ROSA26mT/mG mouse were prepared in a similar manner (excluding the addition of methylene blue). We performed confocal imaging to acquire z-stacks of 7–10 overlapping regions of interests to allow for manual stitching, with 1 μM z-steps (20Χ) or 0.5 μM steps at 40X. We imaged through to the midpoint of the vessel except when imaging the valve interstitial cells, in which case the entire vessel was imaged. Max projections were made using FIJI. Following live imaging, the vessels were pressurized to 5 cmH_2_O and fixed with 4% paraformaldehyde for 30 min at room temperature. IALVs were then washed with PBS containing 0.1% Triton X-100 (PBST) 3 times and blocked for a minimum of 2 hr with Blockaid^®^ (B-10710, ThermoFisher Scientific). IALVs were then stained with the corresponding primary antibodies in BlockAid^®^ Solution: anti-smooth muscle actin (SMA) 1:500 (Sigma, A2547), anti-GFP 1:200 (ThermoFisher, A11122), anti-cKit 1:100 (Cell Signaling, 3074), anti-Vimentin 1:100 (Thermofisher , OMA1–06001), anti-desmin 1:200 (Invitrogen, PA5–16705), anti-GFP 1:200 (Abcam, ab13970, anti-CD34 1:200 (Invitrogen, 14–0341-82), anti-PDGFRΑ 1:200 (R&DSystems, AF1062), anti-PDGFRβ 1:200 (eBiosciences, 14–1402-82), anti-calponin 1:500 (Abcam, AB46794), anti-MYH11 1:500 (Abcam, AB124679), anti-Sca1 1:200 (Biolegend, 108101). IALVs were then washed in PBS and incubated overnight with the corresponding donkey secondary antibodies (ThermoFisher^®^) at 1:200. After a final wash, IALVs were re-cannulated and pressurized for imaging using the aforementioned spinning disk confocal and Hamamatsu Orca Flash4 camera using a 20X air objective (Olympus UplanApo, 0.75) or 40X (Olympus UApo A340, 1.15) water objective. Images were taken as described above, and the resulting stacks were turned into a max projection using FIJI. Colocalization analysis of the max projections of CD34 and PDGFRα was performed using the BIOP JACoP colocalization plugin (Bolte and Cordelieres, 2006) with both Pearson’s and Mander’s coefficients reported.

### LMC Dissociation and FACS Collection-

IALVs vessels *PdgfrαCreER*^*™*^-ROSA26mT/mG, *PdgfrβCreER*^*T2*^-ROSA26mT/mG, *Myh11CreER*^*T2*^-ROSA26mT/mG, Macgreen, and *Prox1-eGFP* mice were dissected and cleaned of excess adventitia and adipose tissue in Krebs buffer. Isolated vessels were then transferred into a low Ca^2+^ PSS solution supplemented with 0.1 mg/mL bovine serum albumin (BSA, Amersham Life Science, Arlington Heights, IL). Primary LMCs were collected by enzymatic dissociation of IALVs. The dissected vessels were cleaned in room temperature Krebs-BSA buffer and then transferred into a 1-mL tube of low-Ca^2+^ PSS on ice, washed, and equilibrated for 10 min. Vessels were then digested in low-Ca^2+^ PSS with 26 U/mL papain (Sigma, St. Louis, MO) and 1 mg/mL dithioerythritol for 30 min at 37°C and were gently agitated every few minutes. This solution was then decanted and replaced with low-Ca^2+^ PSS with containing 1.95 collagenase H (U/mL, Sigma), 1.8 mg/mL collagenase F (Sigma), and 1mg/mL elastase (Worthington LS00635) and incubated for 3 – 5 min at 37° C. The mixture was then spun down at 1000 rpm for 4 min, the digestion buffer removed, and replaced with low-Ca^2+^ PS. This process was repeated twice to remove residual digestion buffer. The vessel was then triturated with a fire-polished Pasteur pipette to dissociate the cells into a single cell suspension, passed through a Falcon cap strainer (35 μm), and resuspended in ice-cold low-Ca^2+^ PSS for sorting. For iCre-ROSA26mT/mG mice, GFP^+^RFP^−^ cells or GFP^+^ cells from Macgreen and *Prox1-eGFP* mice were then FACS purified straight into RNA isolation buffer for RT-PCR analysis. FACs was performed with a Beckamn-Coulter MoFlo XDP instrument using an excitation laser (488 m) and emission filter (530/40 m). Sorting was performed using 70-μm nozzle at a sheath pressure of 45 p.s.i. and sort rate of 100 events/s and with an efficiency of >90%. To maximize cell yield, we isolated both the left and right full-length IALVs vessels from 2 mice for digestions and subsequent FACS collection. For *Myh11CreER*^*T2*^-ROSA26mT/mG and *PdgfraCreER*^*™*^-ROSA26mT/mG, the yield averaged 1000–2000 cells per mouse. For *Prox1-eGFP* mice, LEC yield was typically 1500–2000 cells per mouse.

### RT-PCR Profiling of FACS Purified Cells-

Total RNA was extracted from FACS purified GFP^+^ cells from the isolated IALVs vessels using the Arcturus PicoPure RNA isolation kit (ThermoFisher Scientific, Waltham, MA) per the listed instructions. Prior to elution in 20 μl of water, on-column DNAse digestion (Qiagen, Valencia, CA) was performed to ensure removal of genomic DNA contaminants. RNA was converted into cDNA using SuperScript III First-Strand Synthesis System (Thermo Fisher Scientific, Waltham, MA) using oligo (dT) and random hexamer priming following the manufacturer’s protocol. Each RT reaction used approximately 50–100 cells worth of RNA based on the sorted cells count number. Our PCR reaction mixture contained first-strand cDNA as the template, 2 mM MgCl_2_, 0.25 μM primers, 0.2 mM deoxynucleotide triphosphates; and GoTaq^®^ Flexi DNA polymerase (Promega, Madison, WI). The PCR program comprised an initial denaturation step at 95°C for four min; followed by 35 repetitions of the following cycle: denaturation (94° C, 30 s), annealing (58° C, 30 s), and extension (72° C, 30 s). This was followed by a final elongation step for 5 min at 72° C. PCR amplification products were separated on a 2% agarose gel by electrophoresis, stained with SYBR-Safe (Thermo Fisher Scientific, Waltham, MA), and visualized by UV trans-illumination. All primers were designed to amplify an intron-spanning region. Endpoint RT-PCR Primer sequences, amplicon size, accession numbers, and source are listed in Table 1.

### *Ex vivo* Ca^2+^ imaging with the genetically encoded GCaMP6f Indicator-

*cKitCreER*^*T2*^, *Myh11CreER*^*T2*^, and *PdgfrαCreER*^*™*^ mice were crossed with GCaMP6f mice in a similar manner as described for ROSA26mT/mG. *cKitCreER*^*T2*^-GCaMP6f, *PdgfrαCreER*^*™*^-GCaMP6f, and *Myh11CreER*^*T2*^-GCaMP6f were induced with tamoxifen (10 mg/mL) for 5 consecutive days by i.p. injection. IALVs isolated from *cKitCreER*^*T2*^-GCaMP6f, *PdgfrαCreER*^*™*^-GCaMP6f, and *Myh11CreER*^*T2*^-GCaMP6f were cannulated as described above. The cannulated vessel, with micropipette holders, observation chamber and V-track mounting system, was transferred to the stage of the spinning disk confocal with a Prime95B scMOS camera (Photometrics), a Cascade II EMCCD (Photometrics), or an Ixon888 EMCCD camera (Andor) for Ca^2+^ imaging (Castorena-Gonzalez et al., 2018b). Pressures for the input and output cannula were connected to a T-junction which was set briefly to 8 cmH_2_O and the vessel lengthened to remove axial slack. A peristaltic pump maintained constant perfusion of the observation chamber with Krebs buffer at a rate of 0.5 mL/min while the vessel equilibrated at 37°C for 30–60 min with pressures set to 3 cmH_2_O. Spontaneous contractions were allowed to stabilize over a period of 30 min and then were blunted with 2 μM wortmannin to limit movement associated with contractions during Ca^2+^ imaging. A Windows-based computer was used to digitize the pressure transducer signals and video image of the vessel from a firewire camera at 30–40 Hz (Davis et al., 2012). A custom-written LabVIEW program (National Instruments; Austin, TX) detected the inner diameter of the vessel from the video (Davis et al., 2011). Once contractions were <5 μm in amplitude, Ca^2+^ recordings were made at 20FPS for 20–40 s.

### Ca^2+^ Imaging and Analysis in IALVs Over the Contraction Cycle-

Background noise was determined by using the histogram feature of FIJI in a rectangle in a region of the field of view without sample. This value was subtracted from the entire field of view. In some cases, the vessel movement due to contraction was offset with video stabilization with the FIJI plugin Image Stabilizer. A max projection was used to create non-overlapping ROIs of GCaMP6f^+^ cells for each iCre-GCaMp6f IALV. From these cell ROIs, the “reslice z” function was used to create a pseudo-linescan STMs which were divided by their baseline values to obtain F/F_0_ values for each individual cell. At least 3 cells, except in the case of 1 *cKitCreER*^*T2*^-GCaMp6f IALV, in which only two cells were observed, were analyzed in this manner for each vessel segment. Max projections of the image stack were then used to create non-overlapping cell masks of 3–5 muscle cells per field of view of one vessel. Ca^2+^ traces for those cells contained 5–10 contraction cycles and Ca^2+^ transients and were characterized for peak intensity (expressed as a baseline-referenced ratio, F/F_0_), frequency, and duration in seconds.

### Analysis of Subcellular Ca^2+^ Transients in *Myh11CreER*^*T2*^-GCaMP6f IALVs

For *Myh11CreER*^*T2*^-We performed Ca^2+^ imaging as above in the presence of 1 μM nifedipine to stop the “Ca^2+^ flashes” associated with action potentials (Zawieja et al., 2018) and focus on the subcellular activity at 3 different experimental pressures of 0.5, 2, and 5 cmH_2_O. For this protocol, we used a particle analysis approach to analyze all Ca^2+^ transients in the field of view. Ca^2+^ transients in intact vessels were quantified by particle analysis as previously described (Drumm et al., 2017; Drumm et al., 2019). Movies of Ca^2+^ transients in intact vessels were imported into custom built Volumetry software (version G8d) and background subtracted. Movies were smoothed using a Gaussian filter: 1.5 × 1.5 mM, StdDev 1.0). Raw Ca^2+^ transients were converted to Ca^2+^ particles (PTCLs) using a flood-fill algorithm as previously described (Drumm et al., 2017; Drumm et al., 2019). PTCLs <10 mm^2^ were rejected to facilitate the removal of noise and then the total PTCL area and PTCL count could be tabulated for each recording.

### Light Activation of ChR2 to stimulate Popliteal Collecting Lymphatic Vessel Contractions.

As the IALV has a nearly continuous contractile cycle, we utilized the popliteal vessel for its much slower contraction frequency in the experiments testing our ability to trigger a propagated contraction upon stimulation of the enforced expression of ChR2. Popliteal vessels were isolated from *cKitCreER*^*T2*^-ChR2/tdTomato, *PdgfrαCreER*^*™*^ -ChR2/tdTomato, or *Myh11CreER*^*T2*^-ChR2/tdTomato mice as previously described (Scallan and Davis, 2013), although we intentionally retained some connective tissue and adipose tissue to ensure we had a sufficient population of recombined cells to test in the adventitia layer of the vessel. Contractions were allowed to stabilize over a 30-min equilibration period with pressure set to 3 cmH_2_O. If basal contraction frequency was too high, we applied pinacidil to the bath in 100 nM increments, without exceeding 600 nM, to further slow contraction frequency to around 6 contractions per minute. Pinacidil at sub 1 μM doses can slow contraction frequency without causing overt hyperpolarization of membrane potential (Davis et al., 2020). Supplemental 100 nM doses of pinacidil were applied throughout the experiment to maintain a spontaneous contraction frequency below 6 per minute to allow ample diastolic time for ChR2 stimulation. Throughout this protocol the popliteal was allowed to contract spontaneously to ensure we had not overly inhibited action potentials by the pacemaking cells with pinacidil. Occasionally spontaneous contractions occurred just prior to contractions and could result in a potential false positive so we performed multiple stimulations over a period of 5 – 10 min, typically waiting at least 3 s after any spontaneous contraction before stimulating. Care was made to align the light fiber in such a way that only part of the vessel would be directly illuminated and so target cells of interest would be directly activated by 473 nm light using a Laser diode (Doric LD Fiber Light Source, Quebec, Canada), through an optical probe with a 10-μm tip (Doric, OPT_200_0.22.010). To further limit the excitation field, the optical probe was coated with black acrylic paint using an eyelash brush so that the uncoated opening was ~2–3 μm. With the probe positioned within 5 μm of one side of the vessel wall, the spread of light covered an area ~10–100 μm wide on the back side of the vessel (depending on the diode amplitude setting). Light pulses, 200 ms in length, were triggered by a Grass S9 stimulator (Harvard Apparatus, Holliston, MA) connected to the external TTL input of the laser diode. Pulse amplitude was adjusted between 40–90 mA using the Laser Diode Module Driver (Doric). A contraction was considered to be triggered if it occurred within 50ms of stimulation. We performed photo-stimulation from 2–4 sites within each vessel, with 6–14 stimulations per site. If a photo-stimulation was triggered incidentally after the initiation of a “spontaneous contraction” it was discarded from the analysis. For *Myh11CreER*^*T2*^-ChR2-tdTomato 6 vessels from 3 separate mice were tested. For *PdgfrαCreER*^*™*^-ChR2-tdTomato 6 vessels from 4 separate mice were tested with a max of two vessels per mouse. For *cKitCreER*^*T2*^-ChR2-tdTomato 7 vessels from 3 separate mice were assessed. Diameter was recorded to align photo-activation with the contraction cycle in a custom Labview program.

### Solutions and Chemicals.

Krebs buffer was composed of (in mM): 146.9 NaCl, 4.7 KCl, 2 CaCl_2_, 1.2 MgSO_4_, 1.2 NaH_2_PO_4_•H_2_O, 3 NaHCO_3_, 1.5 NaHEPES, and 5 d-glucose (pH = 7.4 at 37°C). Krebs-BSA buffer was prepared with the addition of 0.5% (w/v) bovine serum albumin (BSA) while Krebs Ca^2+^-free replaced CaCl_2_ with 3mM EGTA. Tamoxifen was dissolved to 10mg/ml in a Safflower Oil-Ethanol (95%–5% v/v) solution with rocking agitation, separated into aliquots, and stored at −20 °C. Wortmannin was dissolved in DMSO to a stock solution of 1 mM. Pinacidil was dissolved in DMSO to a stock concentration of 1 μM. Nifedipine was dissolved in DMSO to a stock concentration of 1 mM. All chemicals were obtained from Sigma (St. Louis, MO), with the exception of BSA (US Biochemicals; Cleveland, OH), MgSO_4_ and NaHEPES (Fisher Scientific; Pittsburgh, PA).

### Statistical Tests

Statistical differences in the isobaric contractile tests for popliteal cLVs isolated from *PdgfrαCreER*^*™*^-Ano1fl/fl, *PdgfrαCreER*^*™*^-Cx45fl/fl, and *PdgfrαCreER*^*™*^-Cav1.2fl/fl mice over the various contractile parameters were assessed via 1) two-way ANOVAs with Tukey’s multiple comparison tests data performed using Prism9 (Graphpad). Data are plotted as mean ± SEM and significance determined at p < 0.05. We used a categorical Chi-squared statistical test for the experiments assessing our ability to trigger a contraction with activation of ChR2 cells. Ca^2+^ PTCL area and frequency was compared using 1-way ANOVA with Tukey’s post-hoc test. Significance was determined at a p value of < 0.05.

## Supplementary Material

Supplement 1

Supplement 2

Supplement 3

Supplement 4

Supplement 5

Supplement 6

Supplement 7

Supplement 8

Supplement 9

Supplement 10

Supplement 11

Supplement 12

Supplement 13

Supplement 14

Supplement 15

## Figures and Tables

**Table 1:** Primer list for RT-PCR

Gene	Strand	Accession #	Sequence(5’-3’)	Siza	Exon	Source
*Prox1*	s	NM_008937	GTA AGA CAT CAC CGC GTG C	218	1	NIH Primer Tool
	as		TCA TGG TCA GGC ATC ACT GG		2	
						
*CD11b (Itgam)*	s	NM_008401	ATG GAC GCT GAT GGC AAT ACC	203	13	MGH Primer Bank ID 668048a1
	as		TCC CCA TTC ACG TCT CCC A		14	
						
*Pdgfra*	s	NM_011058	AGA GTT ACA CGT TTG AGC TGT C	252	8	MGH Primer Bank 26349287a1
	as		GTC CCT CCA CGG TAC TCC T		10	
						-
*Myh11*	s	NM_013607	AAG CTG CGG CTA GAG GTC A	238	33	MGH Primer Bank ID 7305295a1
	as		CCC TCC CTT TGA TGG CTG AG		34	
						
*CD117 (cKit)*	s	NM_021099	CGC CTG CCG AAA TGT ATG ACG	162	21	(Drumm et al., 2018)
	as		GGT TCT CTG GGT TGG GGTTGC		23	
						
*Pdgfrβ*	s	NM_008809	AGC TAC ATG GCC CCT TAT GA	367	16	(Basciani et al., 2004)
	as		GGA TCC CAA AAG ACC AGA CA		19	
						
*CD144 (VE-cadherin)*	s	NM_009868	CTT CCT TAC TGC CCT CAT TGT	313	3	IDT Primer Quest
	as		CTG TTT CTC TCG GTC CAA GTT		5	
						
*Nos3 (eNOS)*	s	NM_008713	ctg cca cct gat cct aac ttg	143	22	IDT Real time primer tool
	as		cag cca aac acc aaa gtc atg		23	
						
*Acta2 (Smooth Muscle Actin)*	s	NM_007392	GAG CTA CGA ACT GCC TGA C	129	7	IDT TaqMan Mm.PT.58.16320644
	as		CTG TTA TAG GTG GTT TCG TGG A		8	
						
*CaV 1.2 exon1b*	s	NM_001159533	ATG GTC AAT GAA AAC ACG AGG ATG		1	(Cheng et al., 2007)
	as		GGA ACT GAC GGT AGA GAT GGT TGC	234	2	
						
*CD34*	as	NM_001111059	GGT ACA GGA GAA TGC AGG TC	119	2	IDT Mm.PT.58.8626728
	s	NM_133654	CGT GGT AGC AGA AGT CAA GT		1	
						
*Cspg4 (Ng2)*	as	NM_139001	CTT CAC GAT CAC CAT CCT TCC	132	5	IDT Mm.PT.58.29461721
	s		CCC GAA TCA TTG TCT GTT CCC		6	
-	-	-	-	-	-	
*Vimentin*	s	NM_011701	CTG TAC GAG GAG GAG ATG CG	249	1	(Li et al., 2016)
	as		AAT TTC TTC CTG CAA GGA TT		3	
						
*Desmin*	s	NM_010043	GTG GAT GCA GCC ACT CTA GC	218	3	MGH Primer Bank ID 33563250a1
	as		TTA GCC GCG ATG GTC TCA TAC		4	
						
*CD146 (Mcam)*	s	NM_023061	CCC AAA CTG GTG TGC GTC TT	220	1	MGH Primer Bank 10566955a1
	as		GGA AAA TCA GTA TCT GCC TCT CC		3	
						
*KLF4*	s	NM_010637	ATT AAT GAG GCA GCC ACC TG	400	1	(Majesky et al., 2017)
	as		GGA AGA CGA GGA TGA AGC TG		3	
						
*Ly6a (Sca1)*	s	NM_001271416	CTC TGA GGA TGG ACA CTT CT	400	2	(Majesky et al., 2017)
	as		GGT CTG CAG GAG GAC TGA GC		4	
						
*Gli1*	s	NM_01029	ATC ACC TGT TGG GGA TGC TGG AT	316	8	(Kramann et al., 2015)
	as		CGT GAA TAG GAC TTC CGA CAG		10	
						
*CD29 (Itgb1)*	s	NM_010578	TCG ATC CTG TGA CCC ATT GC	170	14	NIH Primer Tool
	as		AAC AAT TCC AGC AAC CAC GC		15	
						
*CD105 (Endoglin)*	s	NM_007932	TGA GCG TGT CTC CAT TGA CC	416	11	NIH Primer Tool
	as		GGG GCC ACG TGT GTG AGA A		15	
						
*CD44*	as	NM_009851	CAC CAT TTC CTG AGA CTT GCT	148	19	IDT Mm.PT.58.12084136
	s		TCT GAT TCT TGC CGT CTG C		18	
						
*CD31 (Pecam1)*	s	NM_008816	CTG CCA GTC CGA AAA TGG AAC	218	7	MGH Primer Bank ID 6679273a1
	as		CTT CAT CCA CTG GGG CTA TC		8	
						
*GJC1 (Connexin 45)*	s	NM_008122	GGT AAC AGG AGT TCT GGT GAA	140	2	IDT Mm.PT.58.8383900
	as		TCG AAA GAC AAT CAG CAC AGT		3	
						
*Anoctamin 1 (TMEM16A)*	s	NM_178642	ggc att tgt cat tgt ctt cca g	141	25	IDT Real time primer tool
	as		tcc tca cgc ata aac agc tc		26	
						
*CD45 (Ptprc)*	s	NM_001111316	ATG CAT CCA TCC TCG TCC AC	225	29	NIH Primer Tool
	as		TGA CTT GTC CAT TCT GGG CG		31	

MGH Harvard Primer Bank (Wang and Seed, 2003; Spandidos et al., 2008; Spandidos et al., 2010)

## Impact:

The presence and functionality of proposed pacemaker cells in collecting lymphatic vessels was tested with various mouse Cre models to drive expression of a recombination reporter ROSA26mT/mG, a genetically encoded Ca^2+^ sensor GCaMP6f, or the optogenetic tool channel-rhodopsin2. Lymphatic CD34^+^ adventitial cells co-express PDGFRΑ^+^ while cKit^+^ cells are mast cells; and neither cell type demonstrated pacemaking functionality. *Myh11CreER*^*T2*^ identified lymphatic muscle cells which exhibited pacemaker behaviors such as pressure-dependent calcium events during diastole and propagated contraction induced by optical stimulation of channel-rhodopsin2.

## Abbreviations:

Ano1: Anoctamin 1
ChR2: channel rhodopsin
cLV: collecting lymphatic vessel
IALV: Inguinal Axillary Lymphatic Collecting Vessel
ICC: Interstitial Cell of Cajal
ICLC: Interstitial Cell of Cajal Like Cell
LEC: Lymphatic Endothelial Cell
LMC: Lymphatic Muscle Cell
STDs: Spontaneous transient depolarizations
STMs: Spatio-Temporal Maps
