# Supplementary figures and images for: Cellular characterization of the mouse collecting lymphatic vessels reveals that lymphatic muscle cells are the innate pacemaker cells
Source: bioRxiv. 2025 Jun 25:2023.08.24.554619. Originally published 2023 Aug 26. Preprint. [Version 5] doi: 10.1101/2023.08.24.554619 (PMC10473772; doi:10.1101/2023.08.24.554619)

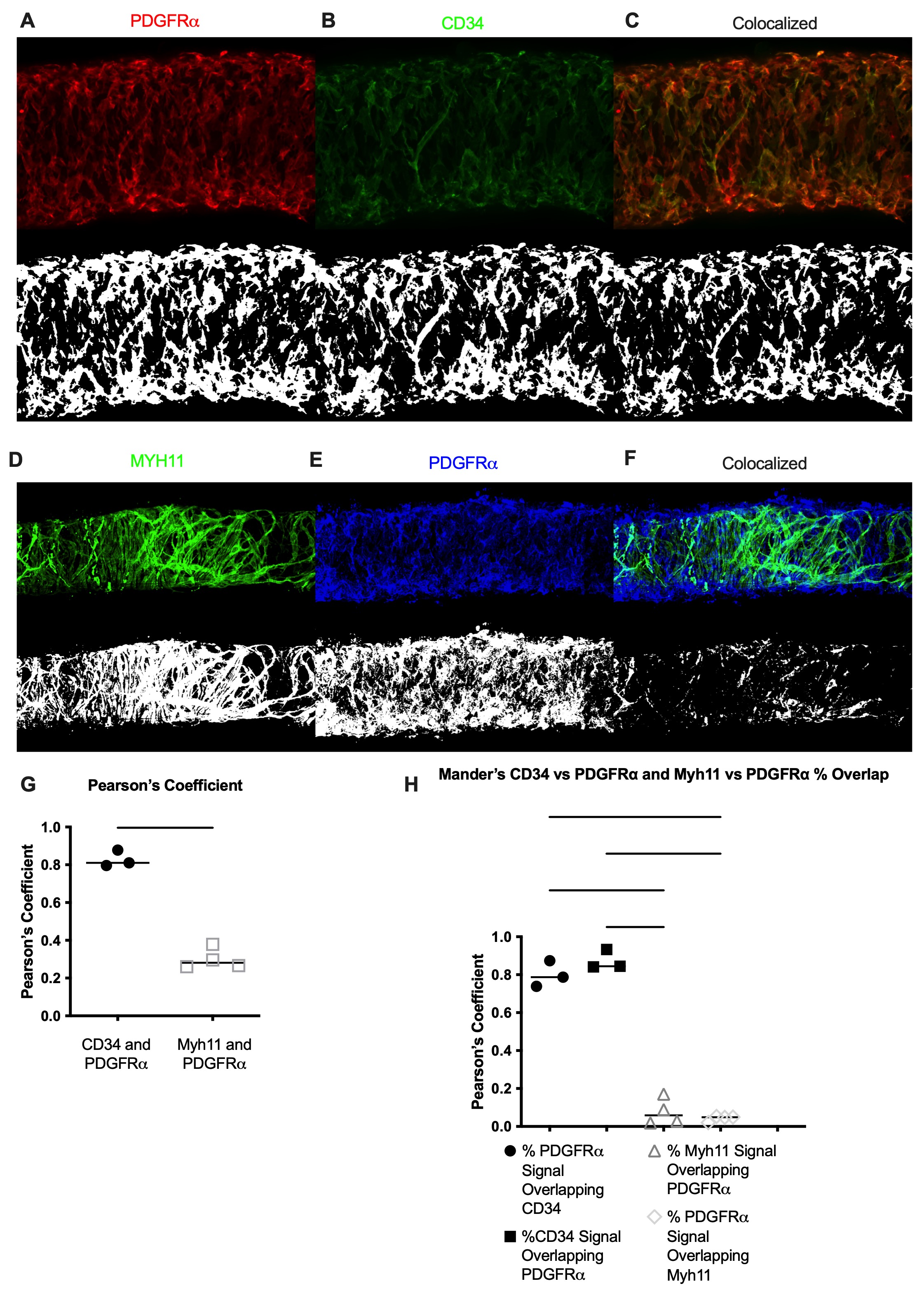

Supplement: Supplement 1 — SuppFigure 1 Colocalization of CD34 and PDGFRα Representative max projections and their corresponding threshold adjusted image for colocalization analysis for PDGFRα (A), CD34 (B), and their colocalized signal (C) and for comparison we tested Myh11 (D) and PDGFRα (E) colocalization (F) using the FIJI BIOP-JACoP colocalization plugin on the z-stacks acquired by confocal microscopy. Pearson’s coefficient (G) and Mander’s coefficients (H) were calculated from n=3 separate stained IALVS, each from a separate mouse for CD34 and PDGFRα and n=4 for Myh11 and PDGFRα. Magnification for A-C 40X and 25x for D-F. Significant differences in colocalization below 0.05 are signified by the overhead lines. [file media-1.jpg]

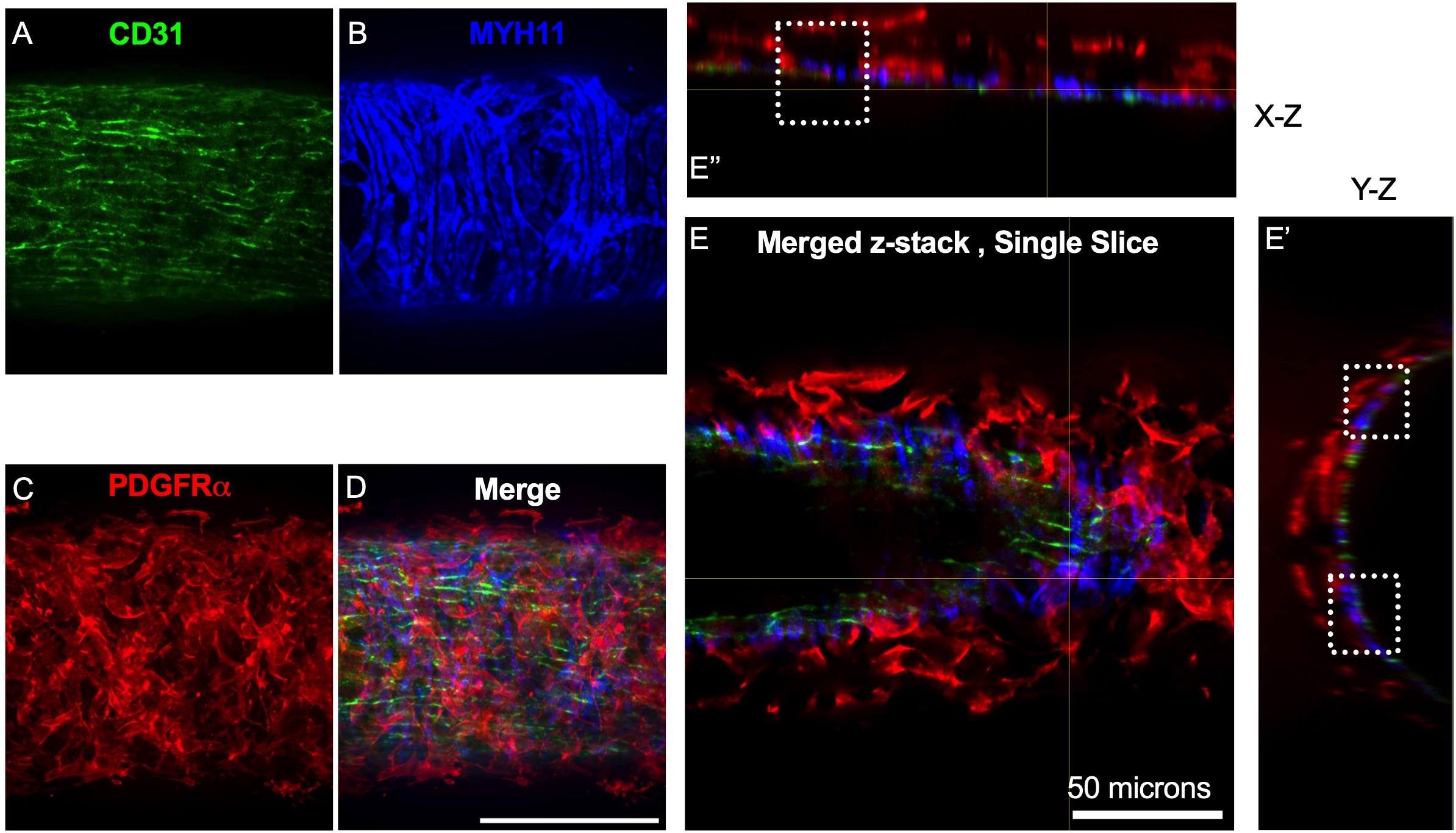

Supplement: Supplement 2 — SuppFigure 2 PDGFRα+ Cells Reside Primarily in the Mouse Lymphatic Collecting Vessel Adventitia and Some in the Subendothelial Space Max projection of confocal imaging of an IALV stained for LECs with CD31 (A), LMCs with MYH11(B), and for PDGFRα (C) with the corresponding merge file (D). Orthogonal views of the z-stack with (E) showing a single slice in the z stack and E’ and E” the orthogonal views. White dotted boxes outline locations where PDGFRα signal is observed between LMC and LEC layers. Scale bar is 100 μm in (D) and 50 μm in (E). [file media-2.jpg]

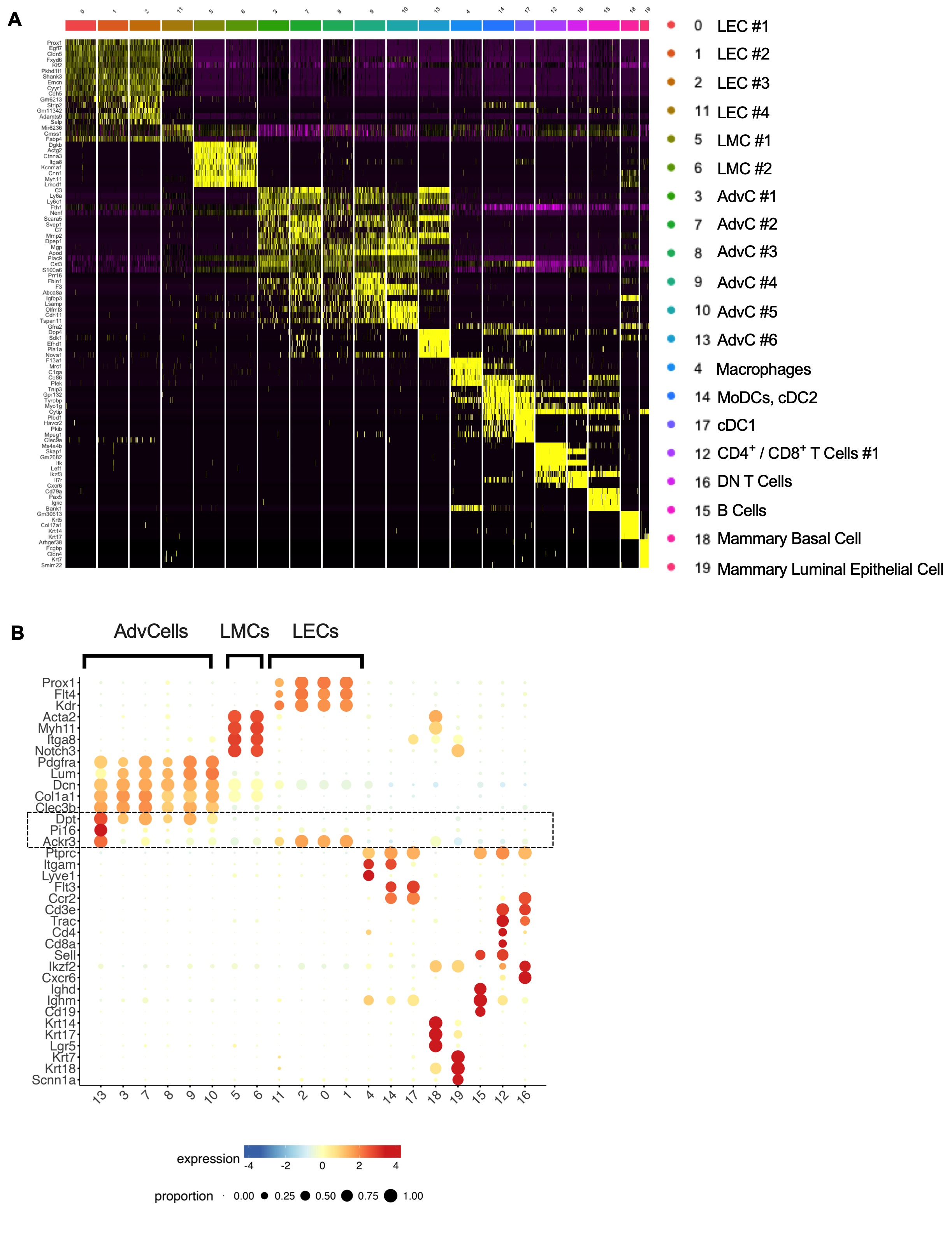

Supplement: Supplement 3 — SuppFigure 3. scRNASeq Analysis of the mouse IALV cell populations. Heatmap of top 4–5 differentially expressed genes, based on p value, for each major cell cluster identified. LECs (Clusters 0,1,2, 11), LMCs (Cluster 5,6), and IALV adventitial cells (AdvC, 3,7,8,9,10,13) were comprised of multiple clusters. B) Bubble plot of common identification genes reveal that the previous reported LMC transcriptome markers Dpt, Pi16, and Ackr3 are specific for a sub population of the Adv and not LMCs. [file media-3.jpg]

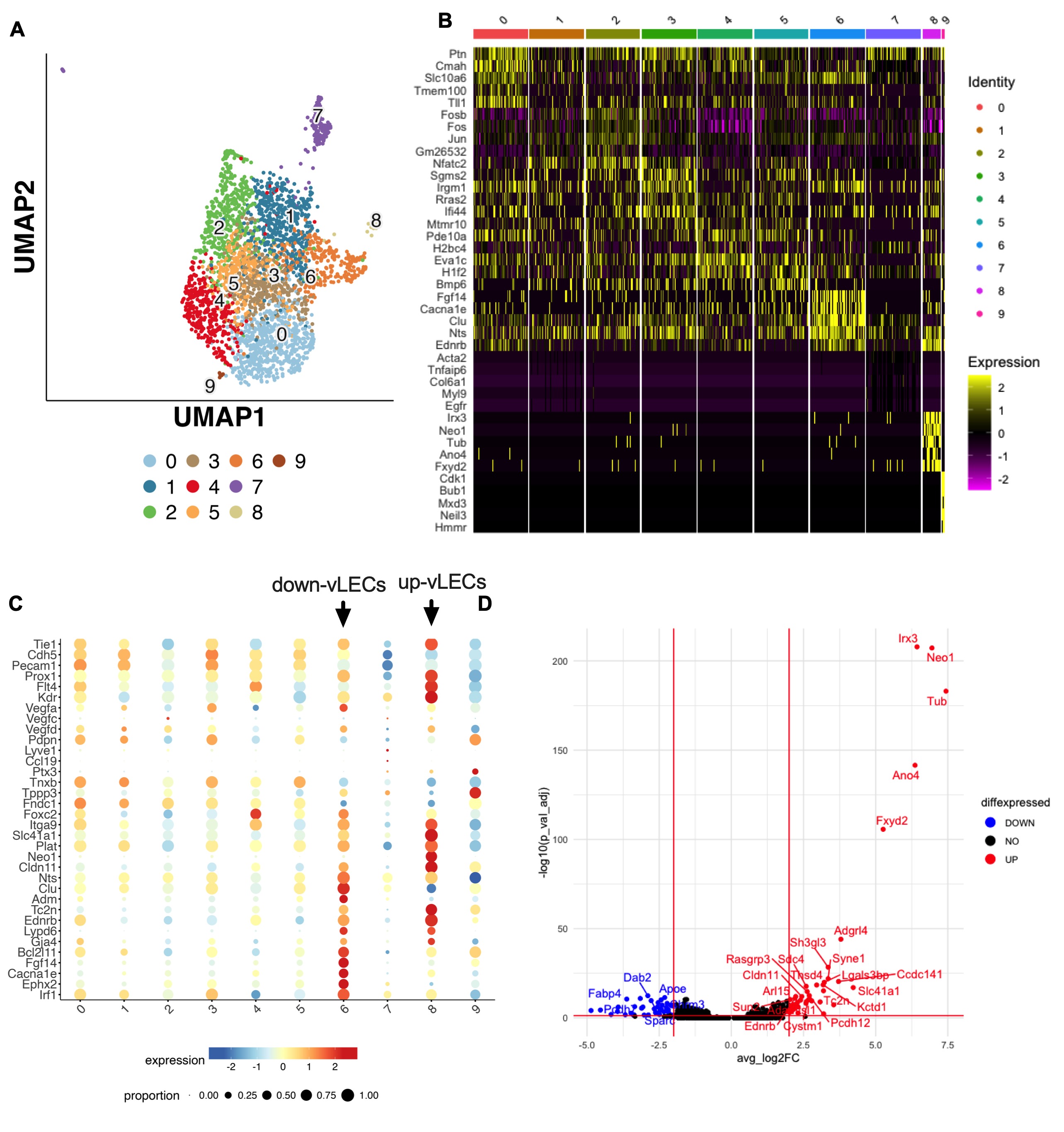

Supplement: Supplement 4 — SuppFigure 4. Subclusters of IALV LECs revealed by scRNAseq. The LECs were further sub-clustered to reveal 10 putative LEC subclusters (0–9) as shown in the UMAP (A) and the top differentially expressed genes amongst those sub-clusters are provided in the adjacent heatmap (B). (C) Bubble plot showing sub-cluster 8 was significantly enriched for previously documented lymphatic endothelial cell up valve genes including Itga9, Cldn11, and Neo1 and Cluster 6 had down valve gene signature including Clu and Adm. The top 30 differentially expressed genes in cluster 8, both positive and negative fold change regulated, are labeled in the volcano plot(D). [file media-4.jpg]

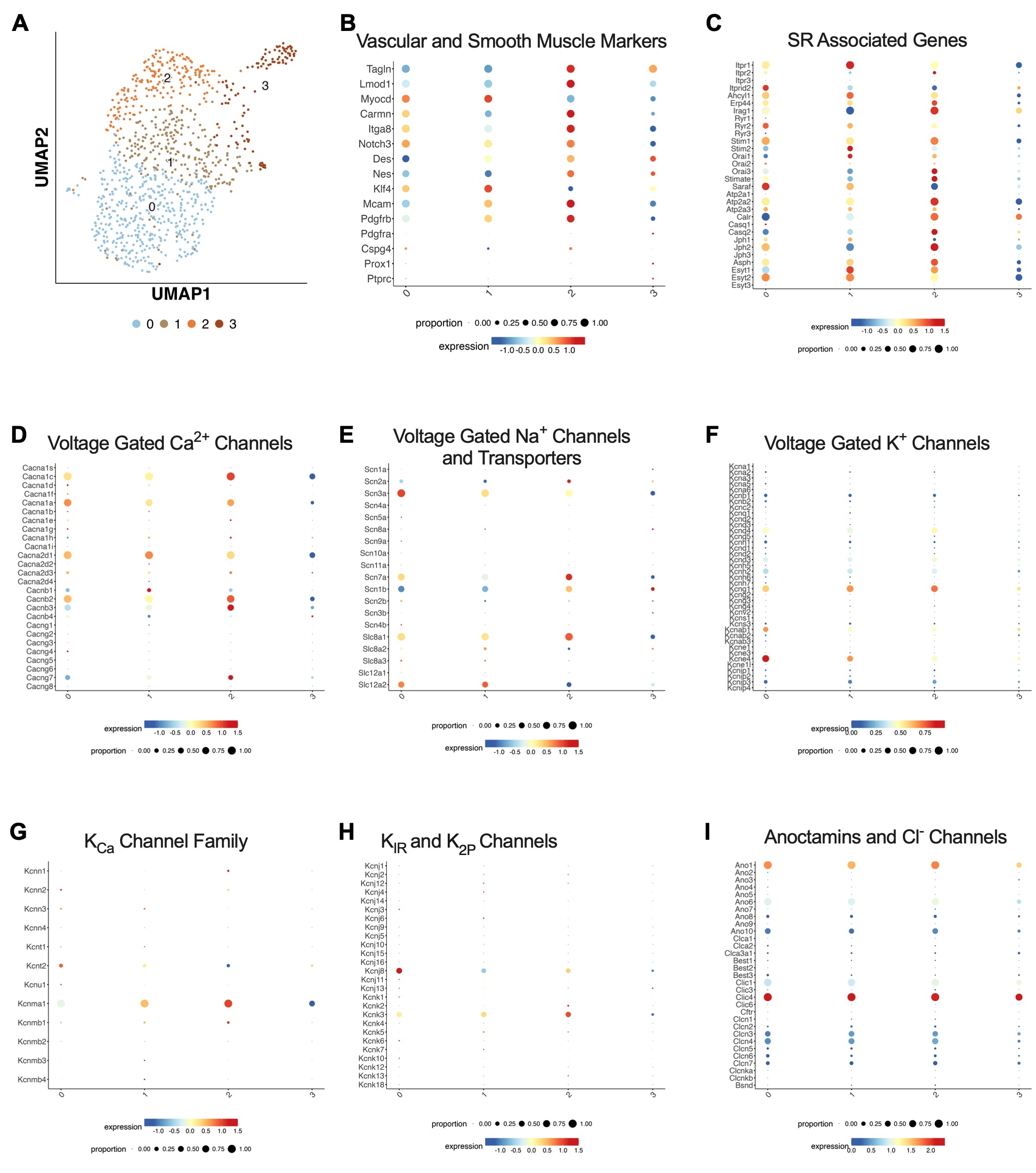

Supplement: Supplement 5 — SuppFigure 5. Subclusters of IALV LMCs revealed by scRNAseq. The LMCs could be subclustered into 4 putative subclusters (0–3) as shown in the UMAP (A). We profiled these subclusters based on their expression of the typical smooth muscle markers (B), SR associated genes (C), voltage gated Ca2+ channels, (D) Voltage gated Na+ channels and Na+ transporters implicated in lymphatic pacemaking (E), voltage gated K+ channels (F), Ca2+ activated K+ channels (G), inward rectifying K+ channels and two-pore domain K+ channels (H), and Cl− channels (I). [file media-5.jpg]

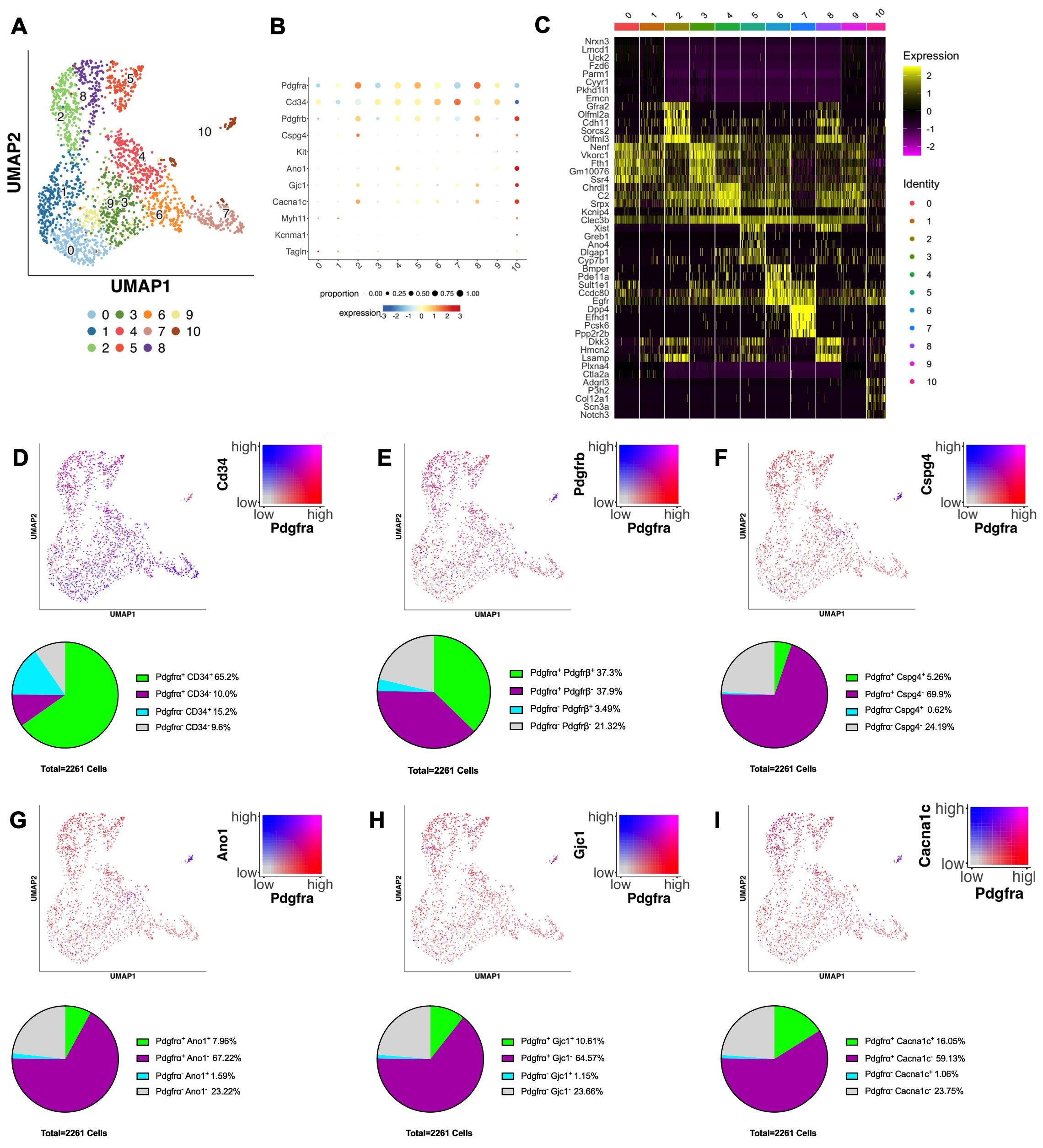

Supplement: Supplement 6 — SuppFigure 6. Subclusters of IALV AdvCs revealed by scRNAseq. AdvCs also could be further subclustered into multiple populations as shown in the UMAP (A). Bubble plot of genes used as Cre drivers and genes associated with pacemaking revealed subcluster 10 had expression of Ano1, Cx45, and Cacna1c (CaV1.2) but with only minimal evidence of LMC contamination as indicated by muscle signature genes Myh11, Kcnma1, and Tagln. C) Heatmap of the top differentially expressed genes among each of the subclusters. We assessed co-expression of Pdgfrα with CD34 (D) to confirm our immunofluorescence imaging (Sugg Figure 1), and assessed the co-expression of Pdgfrα with the pericyte markers Pdgfrβ (E) and Cspg4 (F). We further assessed co-expression of Pdgfrα the genes linked with contractile dysfunction Ano1 (G), Gcj1 (H), and Cacna1c (I) to ensure PdgfrαCreERTM would target the AdvCs expressing these genes. The cyan colored slice of the pie chart indicates the minor population of cells expressing these genes that did not express Pdgfrα. [file media-6.jpg]

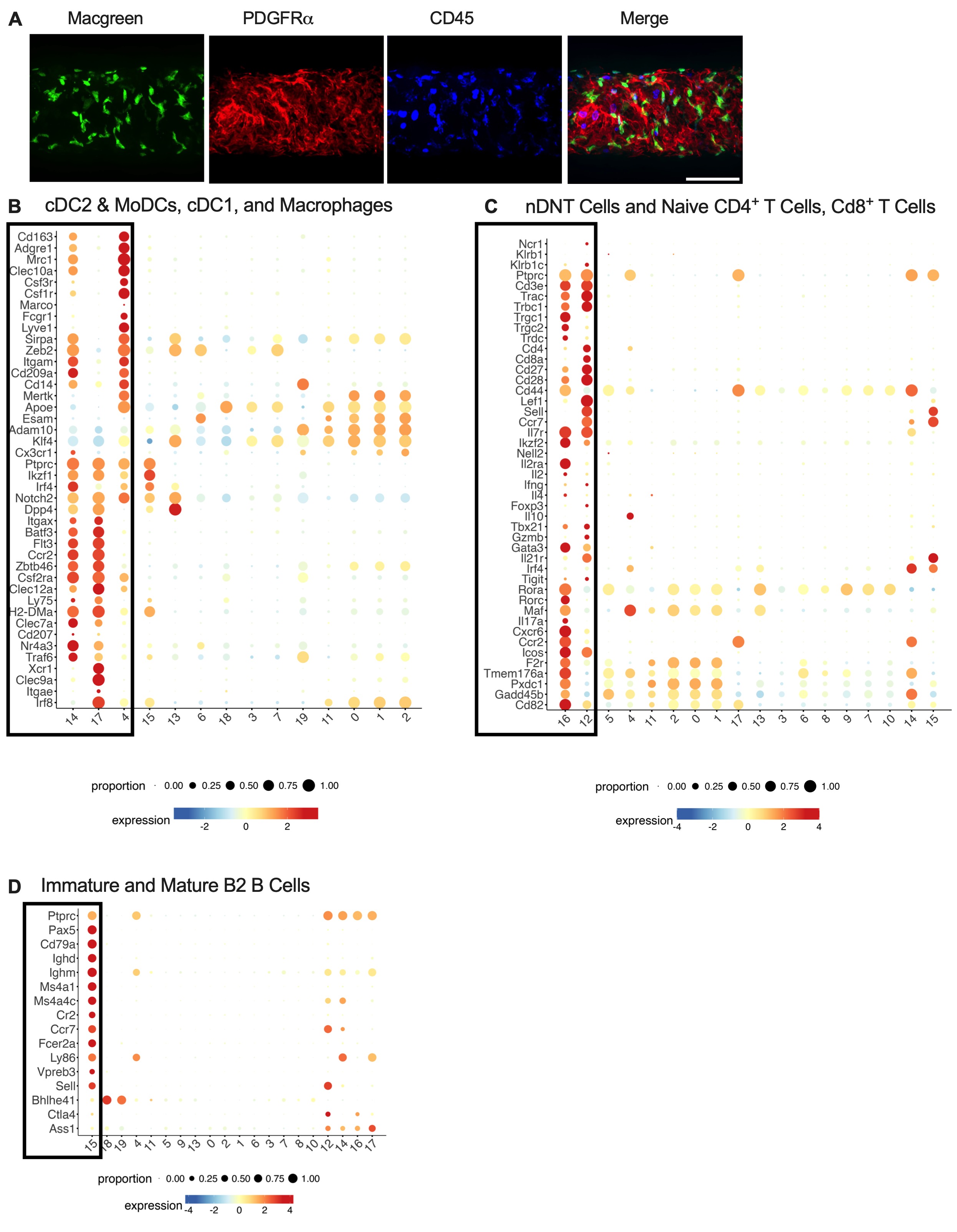

Supplement: Supplement 7 — SuppFigure 7. Immune cell populations associated with the mouse IALV. Lymphatic vessels are host to numerous immune cell populations, including monocyte, macrophage, and dendritic cell populations are revealed by immunofluorescent staining for eGFP in the “Macgreen” (Csf1r-eGFP) reporter mice (A). Staining for Pdgfrα (B) demonstrates that AdvCs are distinct from the GFP+ cells nor do they stain for the hematopoietic marker Ptprc (CD45) (C, D). Bubble plot of our scRNASeq analysis of IALVs revealed macrophages (cluster 4), moDCs (cluster14) and cDC1 cells (17) based off identifying gene markers (B). C) Bubble plot of T-cell markers revealed multiple populations of T cells including naive double negative T-cells (Yang et al., 2021) and naive CD4+ and CD8+ T-cells. A bubble plot for B-cell markers showed that cluster 15 had an expression profile for immature and mature B2 B-cells (D)(Luo et al., 2022b). [file media-7.jpg]

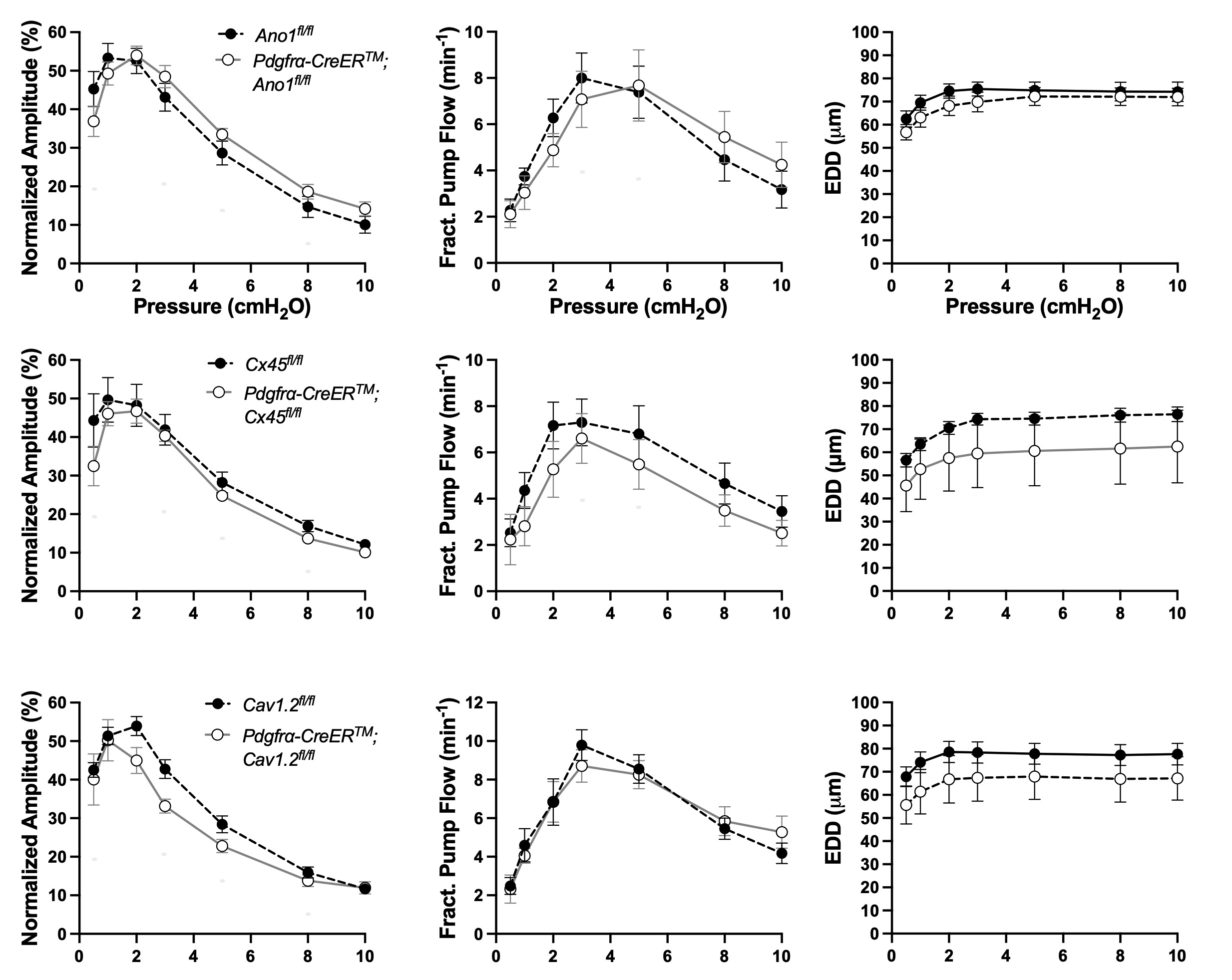

Supplement: Supplement 8 — SuppFigure 8 Contractile indices from isobaric myography on cLVs from PdgfrαCreERTM driven deletion of Ano1, CX45, and CaV1.2 Summary of the contractile parameters recorded from popliteal cLVs in PdgfrαCreERTM-Ano1fl/fl, PdgfrαCreERTM-Cx45fl/fl mice, PdgfrαCreERTM-Cav1.2fl/fl mice. No differences in normalized contraction amplitude (A, D, G), fractional pump flow (B, E, H), or end diastolic diameter (C, F, I) were observed. The contractile data from control Cav1.2fl/fl vessels was previously published but was separated by sex (Davis et al., 2022) while they are combined here. [file media-8.jpg]

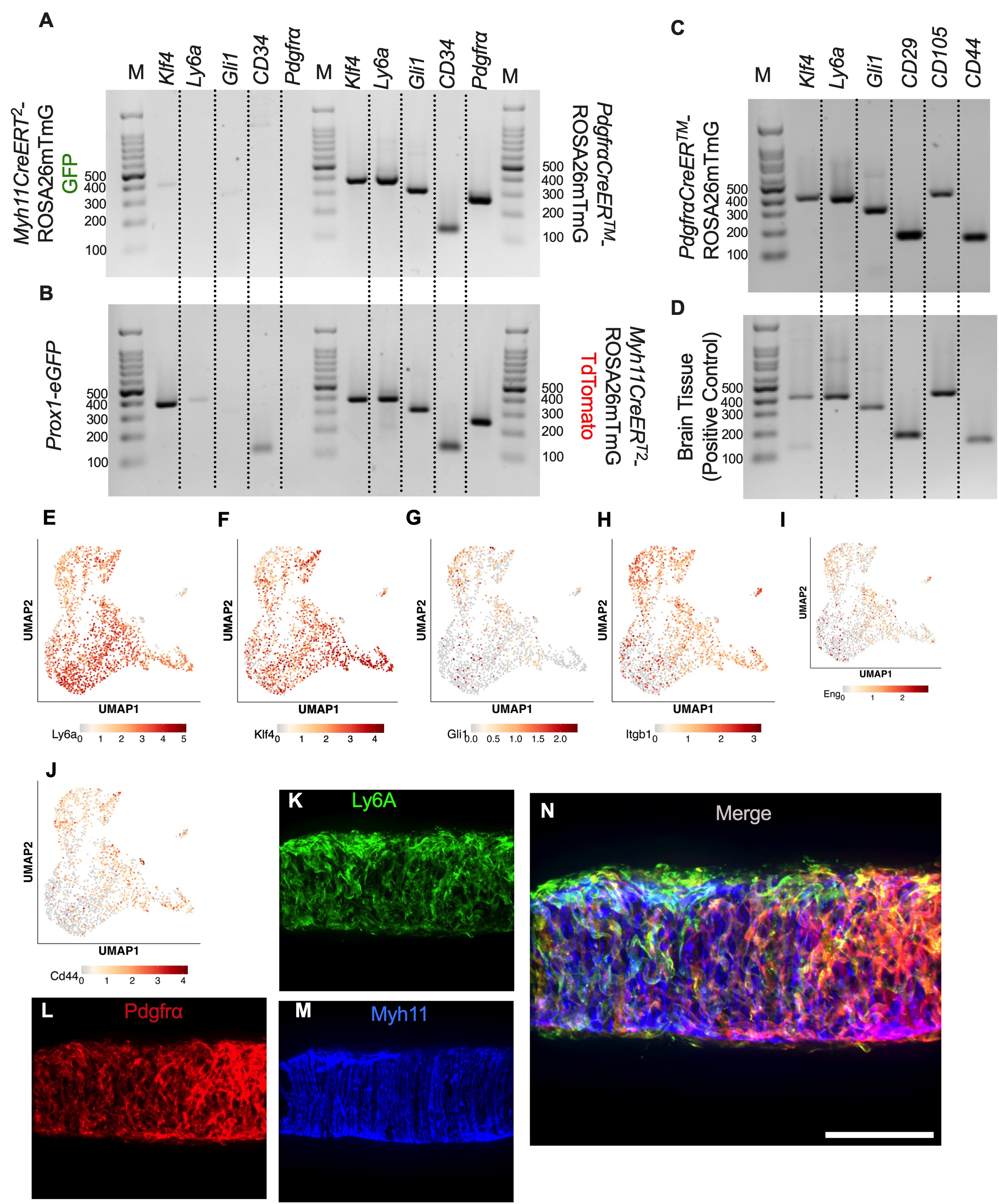

Supplement: Supplement 9 — SuppFigure 9 PDGFRα AdvCs Include Multipotent Cell Representative RT-PCR results profiling purified GFP+ cells purified from IALVs isolated from PdgfrαCreERTM-ROSA26mTmG via FACS. PDGFRα cells expressed the multipotent markers Klf4, Ly6a, Gli1, CD29, CD105, and CD44 (A) with total brain cDNA serving as a positive control (B). Representative RT-PCR results showing lack of expression of some of these markers in the GFP+ cells purified from Myh11CreERT2--ROSA26mTmG (C) or Prox1-eGFP mice, in contrast to the RFP+ population from Myh11CreERT2--ROSA26mTmG mice (D). RT-PCRs were repeated at least 2 times from separate purified cells populations from different mice. Dot plots of only the AdvCs cluster highlights populations of cells that express genes associated with multipotency such as Ly6a (E), Klf4 (F), Gli1 (G), Itgb1 (H, CD29), Eng (I, CD105), CD44 (J). Expression of protein for Ly6a was confirmed with immunofluorescence. Representative max projections of IALVs stained for Ly6a (K), PDGFRα (L), MYH11 (M) and the corresponding merged file (N). Scale bar is 100 μm. [file media-9.jpg]
